# Silver Deposition on Thin Films of Symmetric Long-Chain Dialkylimidazolium-Based Ionic Liquids

**DOI:** 10.3390/molecules31162798

**Published:** 2026-08-11

**Authors:** Alexandre C. P. M. Alves, Luís M. N. B. F. Santos, José C. S. Costa

**Affiliations:** CIQUP, Institute of Molecular Sciences (IMS), Department of Chemistry and Biochemistry, Faculty of Science, University of Porto, Rua do Campo Alegre s/n, P4169-007 Porto, Portugal; alexandrecostapalves@gmail.com (A.C.P.M.A.); lbsantos@fc.up.pt (L.M.N.B.F.S.)

**Keywords:** ionic liquids, vapor deposition, thin films, microdroplets, silver nanoparticles

## Abstract

The formation and stabilization of silver nanoparticles (AgNPs) in thin films of ionic liquids (ILs) based on long-chain alkylimidazolium cations are demonstrated in this work. IL films were prepared by vacuum thermal evaporation using the Knudsen effusion method onto ITO/glass substrates, leading to the formation of micro- and nanosized structures distributed across the surface. The investigated ILs were symmetrical dialkylimidazolium-based systems: [C_7_C_7_im][NTf_2_], [C_8_C_8_im][NTf_2_], and [C_10_C_10_im][NTf_2_]. Increasing the alkyl side-chain length of the imidazolium cation resulted in larger droplet domains. AgNPs were subsequently deposited onto the IL films by sputtering. The formation of AgNPs was confirmed by scanning electron microscopy (SEM), ultraviolet (UV)–visible spectroscopy, and X-ray photoelectron spectroscopy (XPS). The results show that increasing the alkyl side-chain length promotes more effective confinement and stabilization of AgNPs, leading to improved nanoparticle formation and a narrower size distribution. The temporal stability of the Ag-containing IL films was evaluated under both air exposure and inert argon storage, revealing a strong influence of the surrounding atmosphere on nanoparticle evolution. Among the ILs studied, [C_10_C_10_im][NTf_2_] exhibited the most favorable behavior for AgNP formation and stabilization, providing a more stable and homogeneous nanoparticle distribution over time.

## 1. Introduction

**Figure 1:**
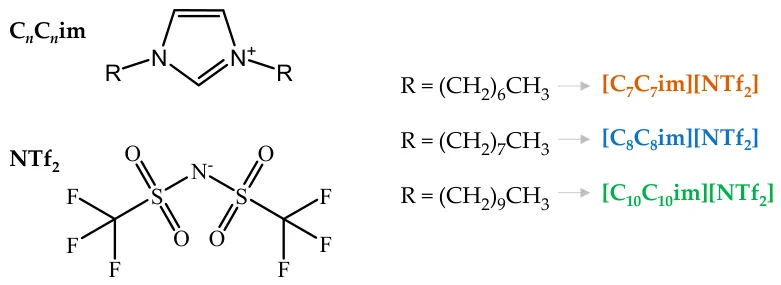
Molecular structures of the ionic liquids investigated in this work.

Ionic liquids (ILs) are commonly defined as compounds composed entirely of ions that melt at relatively low temperatures. Most ILs are characterized by negligible vapor pressure, high thermal stability, wide electrochemical windows, and highly tunable physicochemical properties, such as viscosity, polarity, surface tension, and coordinating ability. These features have attracted considerable interest in a wide range of applications, including catalysis, electrochemistry, separation processes, energy storage, and nanomaterials synthesis [1,2,3,4]. The substantial number of possible cation-anion combinations allows ILs to be designed for different functionalities [5,6]. This structural versatility is particularly important for the synthesis and stabilization of metal nanoparticles (MNPs), as the physicochemical properties of the IL can be tailored to influence nanoparticle nucleation, growth, aggregation, and surface stabilization [7,8,9,10,11,12,13,14,15,16,17]. Indeed, the formation of MNPs in ILs strongly depends on the nature of the IL, since the cations and anions can both simultaneously and individually influence these processes. In the case of cations, the most common ILs are composed of alkyl-substituted imidazolium, pyrrolidinium, pyridinium, or phosphonium-based structures, whereas the anions most frequently reported include bis(trifluoromethylsulfonyl)imide [NTf_2_], tetrafluoroborate [BF_4_], or hexafluorophosphate [PF_6_]. Imidazolium-based ILs are particularly relevant for MNPs synthesis because they can provide electrostatic and steric stabilization through the organization of the cations and anions, forming an interfacial layer around the MNPs [10,14,16]. This interfacial layer acts as a protective shell that reduces nanoparticle aggregation and agglomeration. Another advantage of imidazolium-based ILs is the possibility of tuning the alkyl side-chain length of the cation. This modulates several key physicochemical properties of the IL, including liquid nanostructure, viscosity, surface organization, and metal–IL interactions [11,12,16,17]. Consequently, increasing the alkyl chain length can influence the size, morphology, distribution, and stability of MNPs formed in IL media [9,13]. In a previous study, we investigated the deposition of silver onto thin films of other imidazolium-based ILs. Those depositions focused on the effect of alkyl side-chain length, cation symmetry, and film thickness on the formation of silver nanoparticles (AgNPs). The investigated ILs included [C_4_C_1_im][NTf_2_], [C_2_C_2_im][NTf_2_], and [C_5_C_5_im][NTf_2_], while film thicknesses of 200 ML, 400 ML, 600 ML, and 800 ML (ML = monolayers) were considered. Among these systems, the IL [C_5_C_5_im][NTf_2_] with a film thickness of 800 ML exhibited the most promising behavior for stabilizing smaller AgNPs, showing reduced Ag aggregation on the IL film surface compared to ILs with shorter alkyl chains and thinner films [9]. It was proposed that larger IL droplets, characteristic of thicker IL films and longer alkyl side chains, provide a more favorable medium for the formation, incorporation, and stabilization of AgNPs. These observations motivated the extension of the study to imidazolium ILs with even longer symmetrical alkyl side chains, with the expectation that further enhancement of droplet size and non-polar domain organization could improve AgNP stabilization. Accordingly, the present work investigates the ILs [C_7_C_7_im][NTf_2_], [C_8_C_8_im][NTf_2_], and [C_10_C_10_im][NTf_2_]. The molecular structures of the investigated ILs are shown in Figure 1.

**Figure 2:**
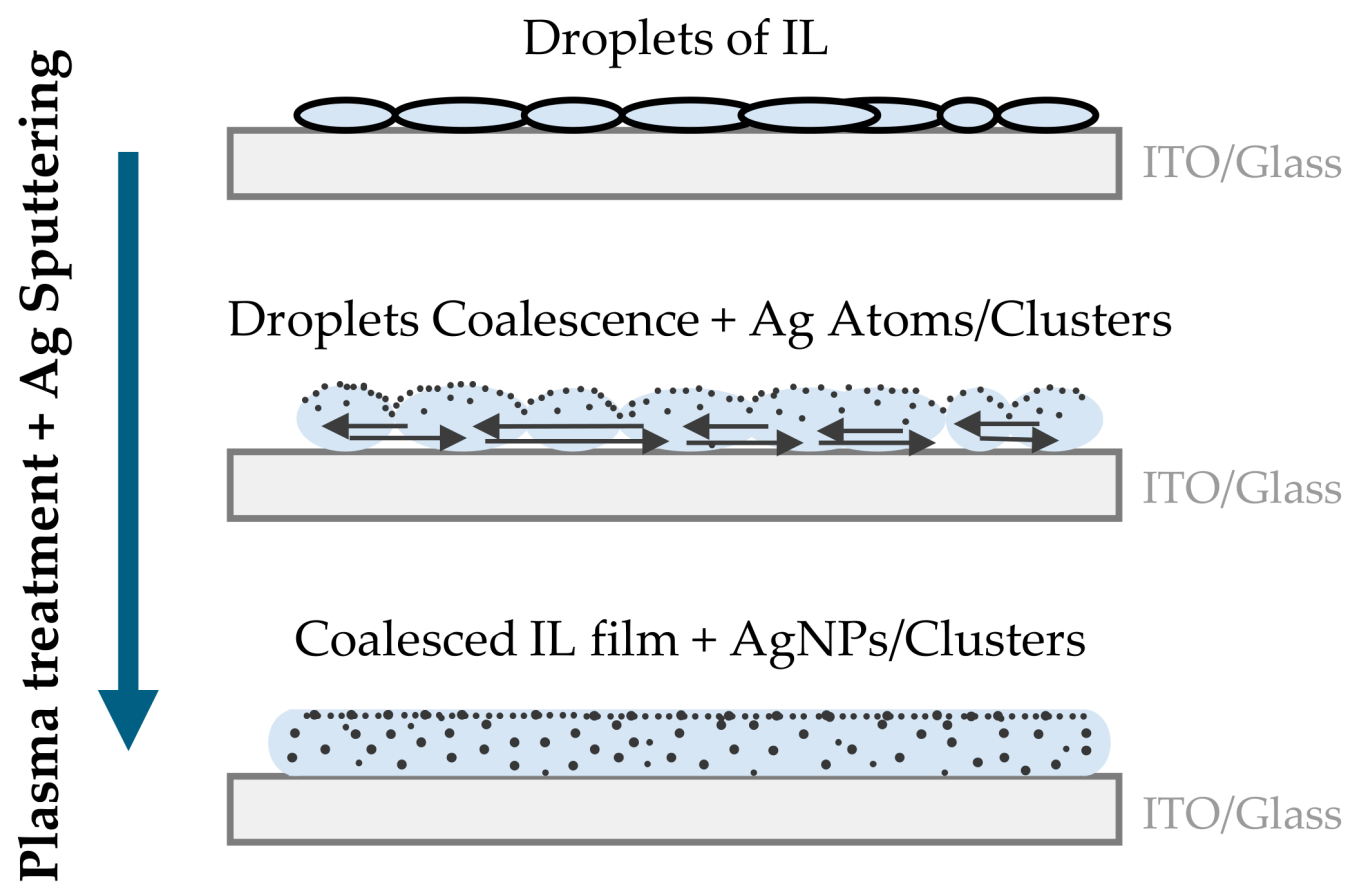
Schematic representation of the silver deposition on ionic liquid films through sputtering.

A step-by-step schematic representation of the Ag deposition process onto the IL films is shown in Figure 2. Representative photographs of the samples after Ag sputtering are provided in Figure S1, where the characteristic yellowish coloration associated with AgNP formation can be observed. The deposition of imidazolium-based ILs onto indium tin oxide (ITO) coated glass substrates by physical vapor deposition (PVD) results in films composed of micro- and nanodroplets rather than fully continuous layers [18]. Depending on the quantity of IL deposited, the nature of the anion, the interaction between the IL and the ITO surface, and the alkyl side-chain length of the imidazolium cation, the morphology of these droplets can change. Increasing the quantity usually leads to larger and more heterogeneous droplets, covering the available substrate surface without fully coalescing to form a continuous film [18]. When IL films of comparable thickness are composed of different cation-anion pairs, the resulting vapor-deposited droplets generally exhibit different sizes and morphologies [9,18,19,20,21,22,23,24,25]. When comparing the morphology of different ILs deposited on identical substrates, the observed differences are mainly governed by the balance between the surface tension of the IL and the IL–substrate interfacial tension, which in turn depends on specific IL–substrate interaction [18,19,20,21,22,23,26,27,28,29,30]. Furthermore, it has been reported that, when the deposited amount and the anion are kept constant, increasing the alkyl side-chain length of the imidazolium cation directly affects the size and morphology of the resulting droplets, leading to larger, more irregular droplets with improved spreading and increased surface coverage [19,20,21,22]. SEM analysis over different time intervals has shown that the IL droplet morphology remains stable after deposition [31]. In fact, alkylimidazolium-based IL microdroplets exhibit high morphological stability under both ambient storage and moderate thermal treatment conditions. In contrast, surface treatments such as argon plasma have a strong effect on the IL morphology and can induce droplet coalescence, leading to the formation of a continuous thin IL layer [9,22].

The sputter deposition of metals onto ILs has emerged as an attractive approach for the synthesis of MNPs in bulk ILs, largely due to their negligible vapor pressure and high stability under vacuum conditions [9,32,33,34,35,36,37,38,39,40,41,42,43,44,45,46,47,48,49,50,51,52,53,54]. These properties allow ILs to be exposed to sputtering environments without significant evaporation or degradation. Most studies in this field have focused on noble metal systems, particularly gold [41,42,48,50,52] and silver nanoparticles [9,39], as well as metallic alloys, due to their technological relevance and well-established applications [32,38,40,43,44,46,47,49,53]. When applied to IL films composed of micro- and nanodroplets, however, sputter deposition using argon plasma has a dual effect on the sample surface, enabling controlled deposition of MNPs while simultaneously inducing the reorganization and coalescence of IL droplets [9]. Smaller, more mobile droplets are expected to reorganize and coalesce more readily under plasma-induced energy input, which may influence the aggregation of metal particles at the IL film surface. In contrast, larger and more viscous droplets require higher energy input to undergo reorganization and coalescence. However, this increased stability allows them to capture and confine MNPs more effectively. Under identical sputtering conditions, such larger IL droplets may therefore promote improved dispersion of MNPs and reduce their aggregation and surface accumulation.

In this context, the present work aims to systematically investigate the formation and stabilization of AgNPs on thin films of symmetrical long-chain imidazolium-based ILs. By combining PVD of ILs with Ag sputter deposition, we explore how increasing alkyl side-chain length affects IL droplet morphology and, consequently, the nucleation, distribution, and stabilization of AgNPs. Particular attention is given to the interplay between IL nanoscale organization and sputtering-induced droplet dynamics, in order to elucidate the mechanisms governing AgNP confinement and aggregation at IL interfaces.

## 2. Results

### 2.1. Morphology of the Ionic Liquid Films

**Figure 3:**
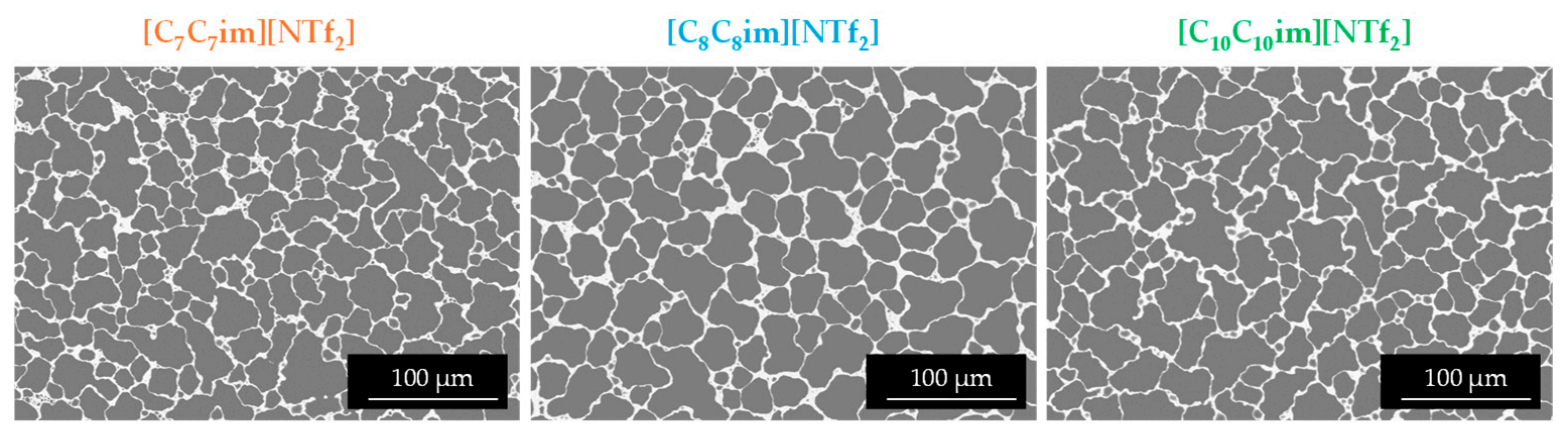
SEM micrographs of [C_7_C_7_im][NTf_2_] (**left**), [C_8_C_8_im][NTf_2_] (**center**), and [C_10_C_10_im][NTf_2_] (**right**) films (400 ML) deposited on ITO/glass substrates. Images correspond to top-view SEM micrographs acquired in backscattered electron (BSE) mode.

The use of imidazolium-based IL films with symmetrical alkyl chains as media for AgNP formation has been previously demonstrated in our earlier work [9]. In that study, [C_5_C_5_im][NTf_2_] was found to promote more efficient AgNP formation and stabilization than [C_2_C_2_im][NTf_2_]. Herein, this approach is extended to ILs with longer symmetrical alkyl chains. The morphology of the IL films deposited onto ITO/glass substrates was investigated by SEM (Figure 3). [C_7_C_7_im][NTf_2_], [C_8_C_8_im][NTf_2_], and [C_10_C_10_im][NTf_2_] were thermally evaporated under controlled conditions using deposition rates of the same order of magnitude (≈0.1 nm·s^−1^, Table S1). Under these conditions, no significant dependence of droplet morphology on deposition rate was observed. This behavior is consistent with previous reports showing that, for these systems, droplet morphology is primarily governed by alkyl chain length, with increasing chain length leading to larger, more spread-out droplets and enhanced surface coverage [19,20,21]. Even at lower deposition rates, ILs with longer alkyl chains, such as [C_8_C_1_im][NTf_2_], [C_8_C_1_im][OTf], and [C_8_py][NTf_2_], have been reported to form droplets with low contact angles and increased spreading on ITO surfaces [19,20,21]. Evaporation and substrate temperatures may also influence droplet coalescence, particularly at higher values. However, all depositions were performed under comparable experimental conditions; therefore, the observed morphological differences can be attributed primarily to the intrinsic properties of each IL. When comparing different ILs, the observed morphological changes are not solely governed by variations in surface tension [55] but instead arise from a combination of IL nanostructuring effects and IL–substrate interfacial interactions. Increasing the alkyl side-chain length enhances nanoscale segregation between polar and non-polar domains, thereby modifying wetting behavior, and is consistent with the experimentally observed formation of larger and more heterogeneous droplets on ITO surfaces [56,57]. In our previous work, we concluded that AgNPs were more effectively stabilized in thicker IL films, whereas thinner films showed a greater tendency for Ag aggregation at the surface [9]. Based on these findings, the present study focuses exclusively on thicker films, namely 400 and 600 ML. The SEM images presented in Figure 3 correspond to films with a thickness of 400 ML, while additional data for 600 ML films are presented in Figure S2. For all investigated ILs, the formation of very large microdroplets was observed, with diameters that, in some cases, exceeded 50 µm. As expected, the 600 ML films exhibit larger and more coalesced structures than the 400 ML films, while maintaining similar overall morphologies across the different ILs (Figure S2). This behavior is characteristic of long-chain ILs and contrasts with that of shorter-chain analogs, which typically form smaller and more spherical droplets [18,19,20,21,22]. Although these large structures exhibit very low contact angles and extensive spreading, complete wetting of the substrate is not achieved, and the films remain composed of discrete micro- and nanodroplets rather than continuous layers (Figure S3). This suggests that on ITO/glass, the IL–IL cohesive interactions remain more favorable than the IL–ITO interactions, leading to a Volmer–Weber-type growth mode [18,58]. In comparison with previous works, the investigated ILs possess significantly longer alkyl side chains, reaching up to twice the length of those previously studied [9]. Consequently, even the 400 ML and 600 ML films examined here exhibited larger droplets than those previously observed for thicker IL films, including 800 ML films of [C_5_C_5_im][NTf_2_]. This behavior further highlights the strong influence of alkyl side-chain length on the morphology of vapor-deposited IL films.

### 2.2. Silver Sputtering onto the Ionic Liquid Films

Following the morphological characterization of the IL films deposited on ITO, silver was deposited onto the samples by sputtering. The morphology and evolution of the AgNPs were primarily investigated by SEM and supported by UV–Vis spectroscopy, whereas XPS was employed as a complementary surface-sensitive technique to investigate the surface chemical composition before and after Ag deposition and during aging.

**Figure 4:**
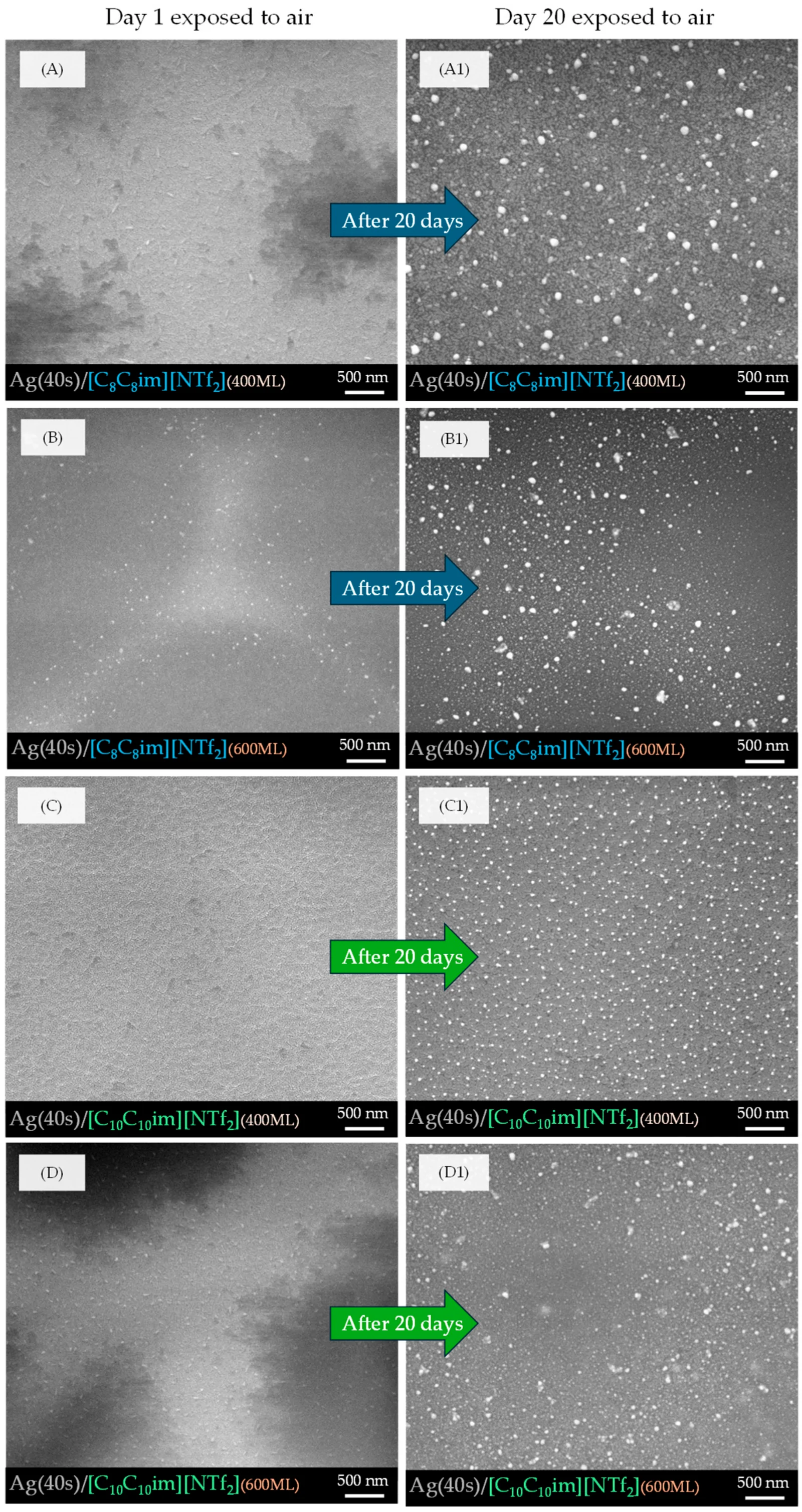
Time-dependent study of the surface morphology of IL films of [C_8_C_8_im][NTf_2_] and [C_10_C_10_im][NTf_2_] after Ag deposition. Morphology of [C_8_C_8_im][NTf_2_] (400 ML) after air exposure for 1 day (**A**) and 20 days (**A1**). Morphology of [C_8_C_8_im][NTf_2_] (600 ML) after air exposure for 1 day (**B**) and 20 days (**B1**). Morphology of [C_10_C_10_im][NTf_2_] (400 ML) after air exposure for 1 day (**C**) and 20 days (**C1**). Morphology of [C_10_C_10_im][NTf_2_] (600 ML) after air exposure for 1 day (**D**) and 20 days (**D1**). SEM images correspond to top-view micrographs acquired in secondary electron (SE) mode.

**Figure 5:**
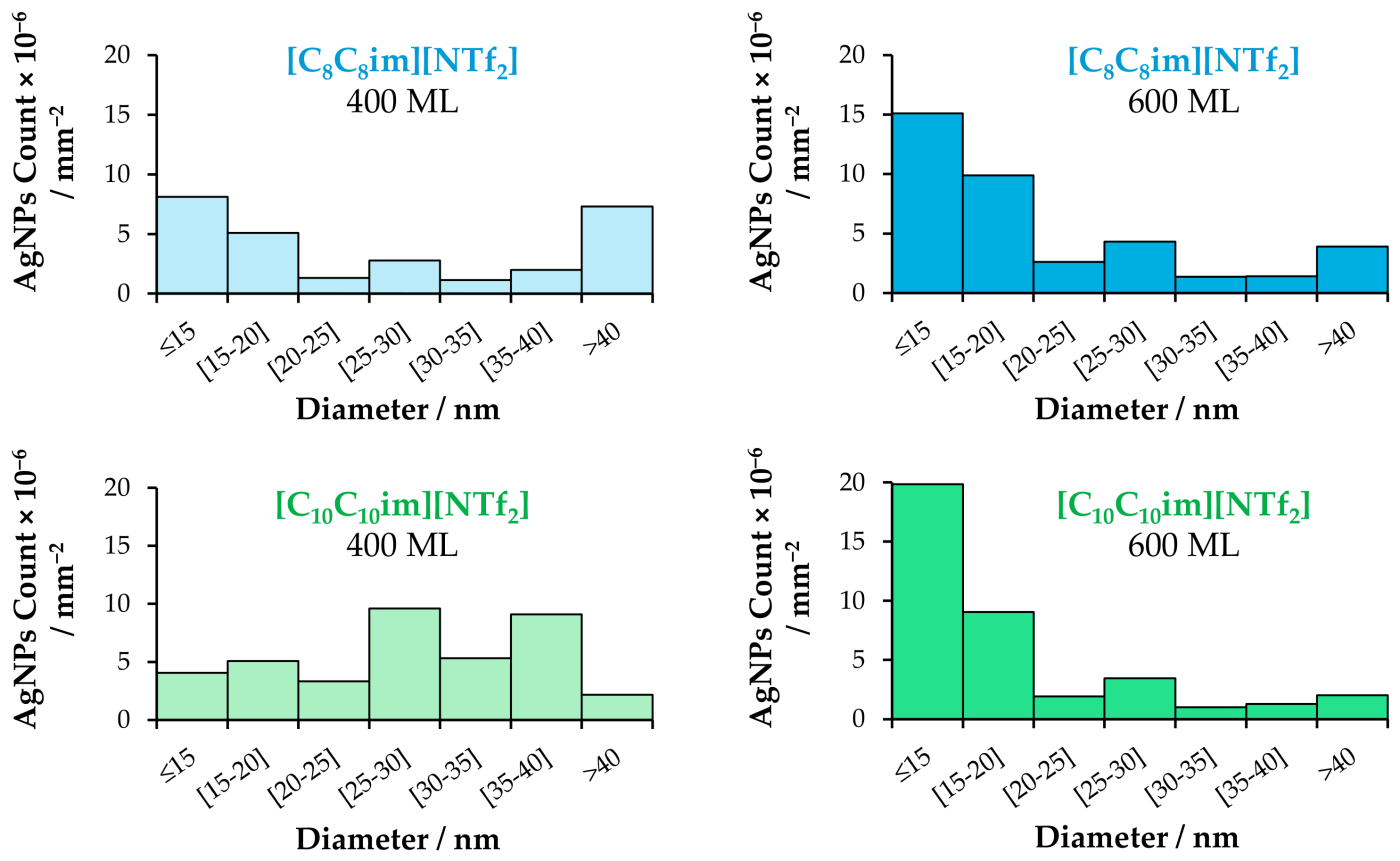
Silver particle count on the surface of [C_8_C_8_im][NTf_2_] and [C_10_C_10_im][NTf_2_] films with different thicknesses (400 and 600 ML) after 20 days of air exposure.

The surface morphology after Ag deposition is shown in Figure 4 for [C_8_C_8_im][NTf_2_] and [C_10_C_10_im][NTf_2_] films with thicknesses of 400 and 600 ML. After 40 s of sputtering (selected based on previous optimization studies identifying this condition as providing the most favorable AgNP formation and distribution), and under identical discharge current conditions, the morphology of the IL films changed markedly. The initial morphology of the IL film changed significantly, indicating extensive droplet coalescence and substantial reorganization of the IL film induced by plasma exposure. The samples exhibited a visible yellowish coloration (Figure S1), which is characteristic of the surface plasmon resonance of AgNPs and provides a first qualitative indication of nanoparticle formation. After sputtering, the samples were exposed to air and analyzed by SEM after 1 and 20 days. The exposure to air was intentionally used to assess the stability and time-dependent evolution of AgNPs confined in IL films under ambient conditions. On the first day of exposure, a thin layer of AgNPs was observed on the surface of all samples, as shown in Figure 4A–D. After 20 days of air exposure, significant morphological evolution was observed. Well-defined bright particles were visible in all samples, indicating an aging-induced reorganization of the AgNPs. In addition, larger spherical particles and aggregates appeared, as shown in Figure 4(A1)–(D1). Among the samples in Figure 4, the 400 ML film of [C_10_C_10_im][NTf_2_] exhibited one of the most homogeneous distributions of small bright particles after 20 days of exposure (Figure 4(C1)). The corresponding 600 ML film also showed an increase in the number of visible particles, although the spatial distribution appeared less homogeneous in certain regions (Figure 4(D1)). Preliminary results obtained for [C_7_C_7_im][NTf_2_], based on SEM observations and UV–Vis analysis, indicated a lower tendency for AgNP formation and stabilization compared to the longer-chain analogs [C_8_C_8_im][NTf_2_] and [C_10_C_10_im][NTf_2_]. Furthermore, the identification and quantification of nanoparticles by SEM proved particularly challenging for these samples due to the low density and limited contrast of the observed features. For this reason, the detailed SEM analyses presented here were focused primarily on [C_8_C_8_im][NTf_2_] and [C_10_C_10_im][NTf_2_], which exhibited a more pronounced nanoparticle population and allowed a more reliable statistical evaluation. The SEM images of the 400 and 600 ML films of [C_8_C_8_im][NTf_2_] and [C_10_C_10_im][NTf_2_] after Ag sputtering and 20 days of air exposure were analyzed using *ImageJ* (version 1.52a) [59], enabling quantification of the particle size distribution at the surface of each sample. The results are summarized in Figure 5. For the 400 ML films, a broad size distribution was observed, with clear differences between [C_8_C_8_im][NTf_2_] and [C_10_C_10_im][NTf_2_]. The former exhibited a more bimodal distribution with populations of small and large particles, whereas the latter showed a distribution centered at intermediate particle sizes. For the thicker (600 ML) films, smaller particle diameters were generally favored, with a higher prevalence of nanoparticles having diameters below 15 nm, suggesting enhanced confinement and stabilization of AgNPs within the thicker IL films.

### 2.3. UV-Vis Analysis of Ag-Containing IL Films

**Figure 6:**
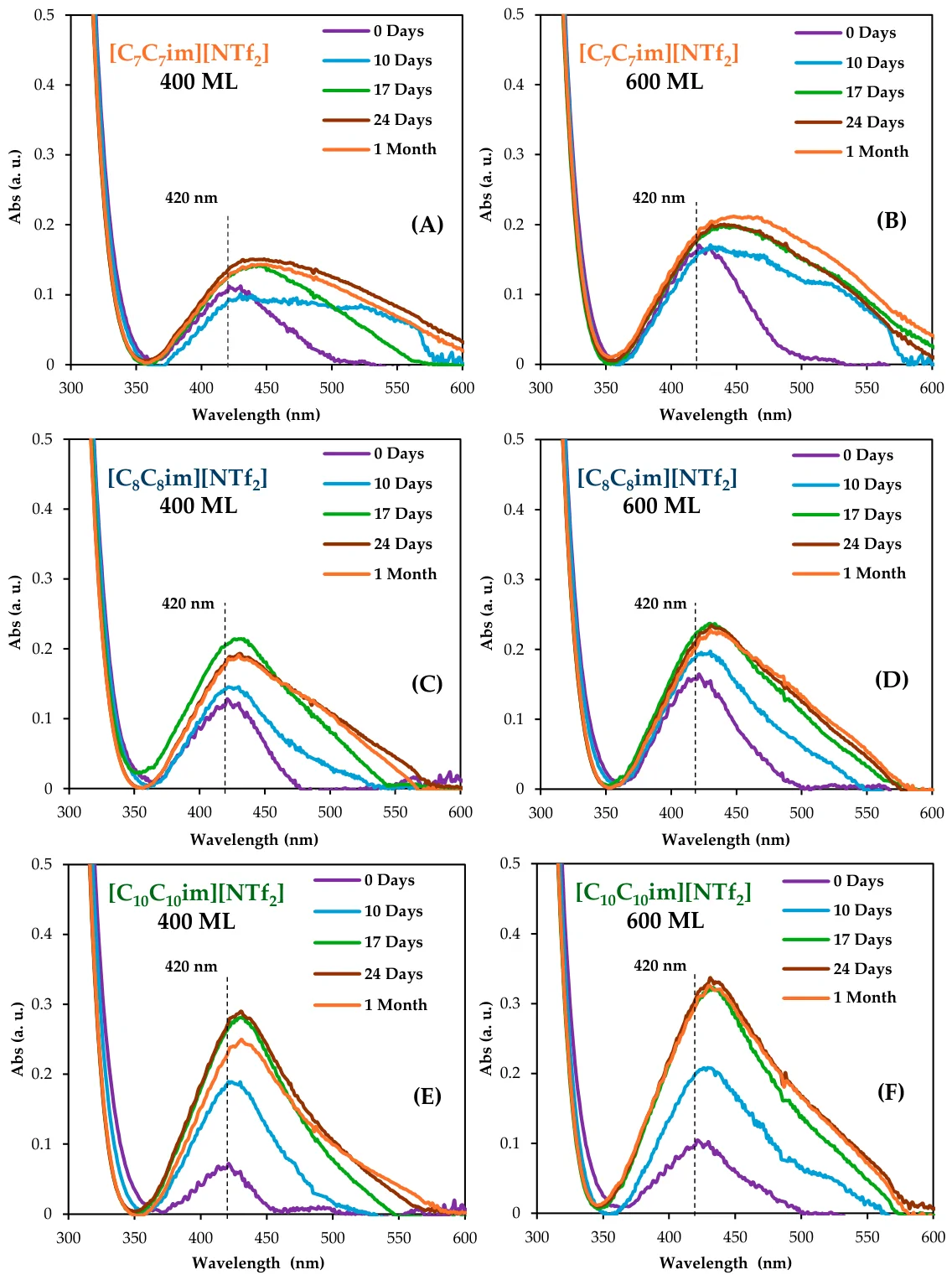
UV–Vis absorption spectra of [C_7_C_7_im][NTf_2_] (**A**,**B**), [C_8_C_8_im][NTf_2_] (**C**,**D**), and [C_10_C_10_im][NTf_2_] (**E**,**F**) films treated with argon plasma and Ag particle deposition (40 s). Spectra are shown for films with thicknesses of 400 (left panels) and 600 ML (right panels), recorded after 0, 10, 17, 24 days, and 1 month of air exposure, highlighting the evolution of the surface plasmon resonance (SPR) band.

The samples were analyzed by UV–Vis spectroscopy immediately after preparation and after 10, 17, 24, and 30 days of exposure to air. This technique was used to evaluate the optical response associated with the formation of AgNPs and the temporal evolution of the samples [60]. The presence of a surface plasmon resonance (SPR) band around 420 nm supports the formation of AgNPs on the IL films studied in this work, as shown in Figure 6 for all samples. It is noteworthy that the position, intensity, and width of these bands vary depending on the IL structure, film thickness, and aging time. In general, the intensity of the SPR band increased during the first days after preparation, reaching a maximum or near plateau between 17 and 30 days, depending on the sample. The [C_7_C_7_im][NTf_2_] film with 600 ML (Figure 6B) exhibited a higher SPR band intensity than the 400 ML (Figure 6A) film when samples at the same aging time were compared. In addition, with increasing aging time, the SPR bands became broader, and a shoulder at higher wavelengths became visible after aging. In the case of [C_8_C_8_im][NTf_2_], the SPR bands also increased in intensity with film thickness (Figure 6C,D). Although a slight red shift and band broadening were observed after air exposure, these effects were less pronounced than for [C_7_C_7_im][NTf_2_], suggesting a more defined optical response associated with AgNP formation. Finally, the [C_10_C_10_im][NTf_2_] films (Figure 6E,F) exhibited the most intense and well-defined SPR bands among all investigated ILs. Both thicknesses showed a clear increase in band intensity from day 0 to later aging times. At the same time, the bands remained sharper than those observed for the other two ILs, and the red shift and broadening were less pronounced. For the 400 ML sample (Figure 6E), the SPR band intensity slightly decreased after reaching a maximum at day 17, whereas the 600 ML sample (Figure 6F) maintained a high intensity up to 30 days. Overall, the UV–Vis spectra confirm the formation of AgNPs in all IL films. The longer-chain ILs, particularly [C_10_C_10_im][NTf_2_], produced more intense and sharper SPR bands, suggesting enhanced stabilization of AgNPs.

### 2.4. UV–Vis Maximum Absorbance and SPR Peak Position

**Figure 7:**
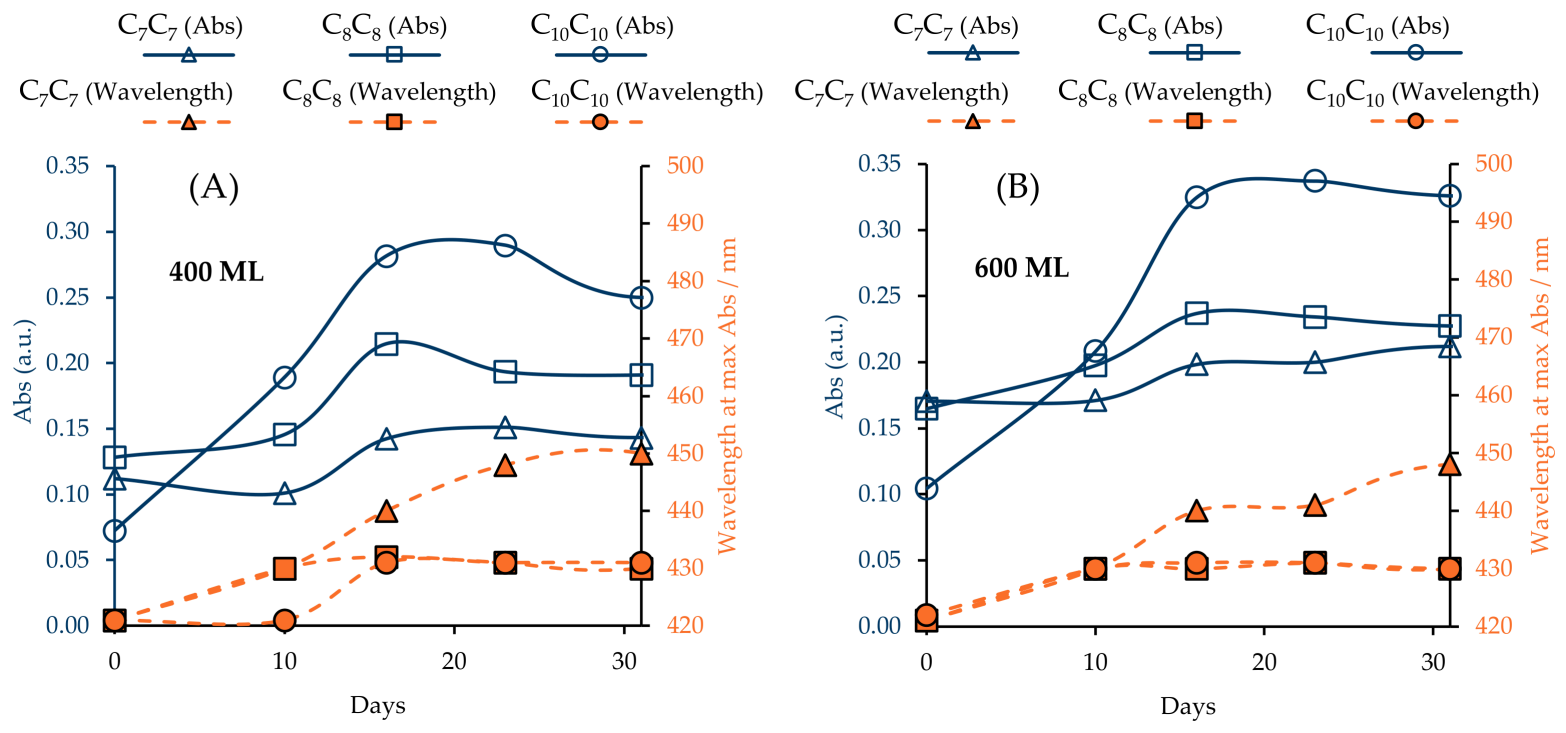
(**A**) Maximum absorbance of the SPR band for IL films of [C_7_C_7_im][NTf_2_], [C_8_C_8_im][NTf_2_], and [C_10_C_10_im][NTf_2_] with 400 ML thickness after 40 s of Ag sputtering, monitored over 31 days (left axis). Corresponding wavelength of maximum absorbance, illustrating the SPR peak shift over the same period (right axis). (**B**) Maximum absorbance of the SPR band for IL films of [C_7_C_7_im][NTf_2_], [C_8_C_8_im][NTf_2_], and [C_10_C_10_im][NTf_2_] with 600 ML thickness after 40 s of Ag sputtering, monitored over 31 days (left axis). Corresponding wavelength of maximum absorbance, illustrating the SPR peak shift over the same period (right axis).

Figure 7 summarizes the evolution of the maximum absorbance of the SPR band and the corresponding wavelength of maximum absorbance for the samples studied. For the 400 ML films (left panel), [C_10_C_10_im][NTf_2_] exhibited the highest absorbance values among the three ILs throughout the aging period. The absorbance increased from day 0 to approximately day 17–24, followed by a slight decrease after 30 days. The corresponding maximum wavelength remained within the 420–430 nm region, with only minor variations over time. The [C_8_C_8_im][NTf_2_] 400 ML film showed intermediate absorbance values, higher than those of [C_7_C_7_im][NTf_2_] but lower than those of [C_10_C_10_im][NTf_2_]. The wavelength of maximum absorbance remained close to that observed for [C_10_C_10_im][NTf_2_], indicating a similar SPR peak position but lower intensity. In contrast, the [C_7_C_7_im][NTf_2_] 400 ML film exhibited the lowest absorbance values and a more pronounced shift in the wavelength of maximum absorbance over time. For the 600 ML films (right panel), a similar trend was observed. The [C_10_C_10_im][NTf_2_] film again showed the highest absorbance values, increasing until approximately day 17 and remaining nearly constant thereafter. The [C_8_C_8_im][NTf_2_] film displayed intermediate values, whereas [C_7_C_7_im][NTf_2_] consistently showed the lowest absorbance after aging. The wavelength of maximum absorbance for [C_10_C_10_im][NTf_2_] and [C_8_C_8_im][NTf_2_] remained relatively stable, while [C_7_C_7_im][NTf_2_] exhibited a progressive red shift up to the final measurement. Overall, Figure 7 demonstrates that increasing the alkyl side-chain length from C7 to C8 and C10 leads to higher SPR absorbance values and improved stability of the SPR peak position over time.

### 2.5. Ionic Liquid Film Surface Morphology After Ag Sputtering

**Figure 8:**
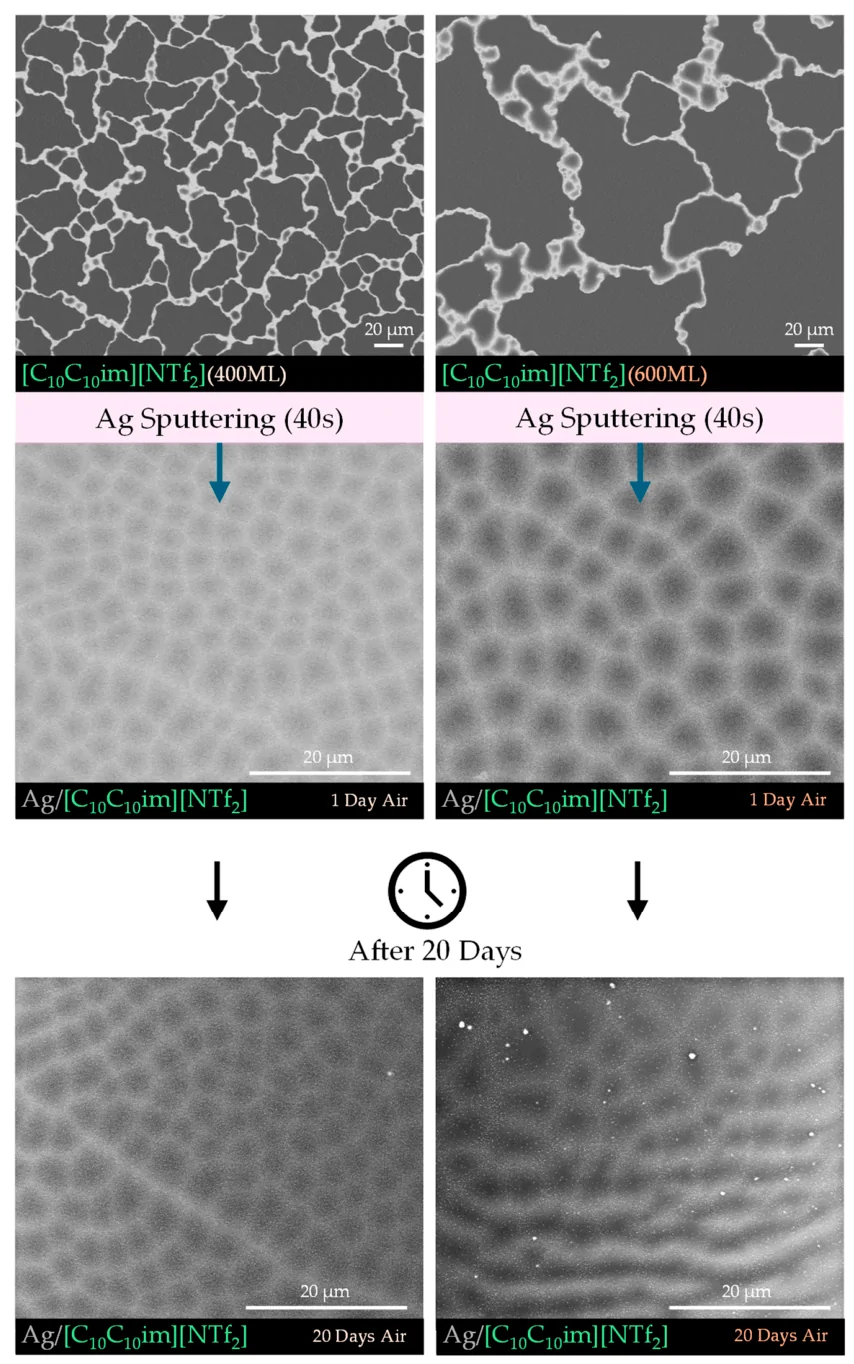
Morphological evolution of [C_10_C_10_im][NTf_2_] films with thicknesses of 400 (**left**) and 600 ML (**right**). Top images correspond to the films after IL thermal evaporation, showing the formation of nano- and microdroplets on the ITO substrate. Center images show the same films after Ag sputtering for 40 s at a discharge current of 20 mA, followed by 1 day of air exposure. Bottom images correspond to the same films after 20 days of air exposure. SEM images correspond to top-view micrographs acquired in backscattered electron (BSE) mode.

**Figure 9:**
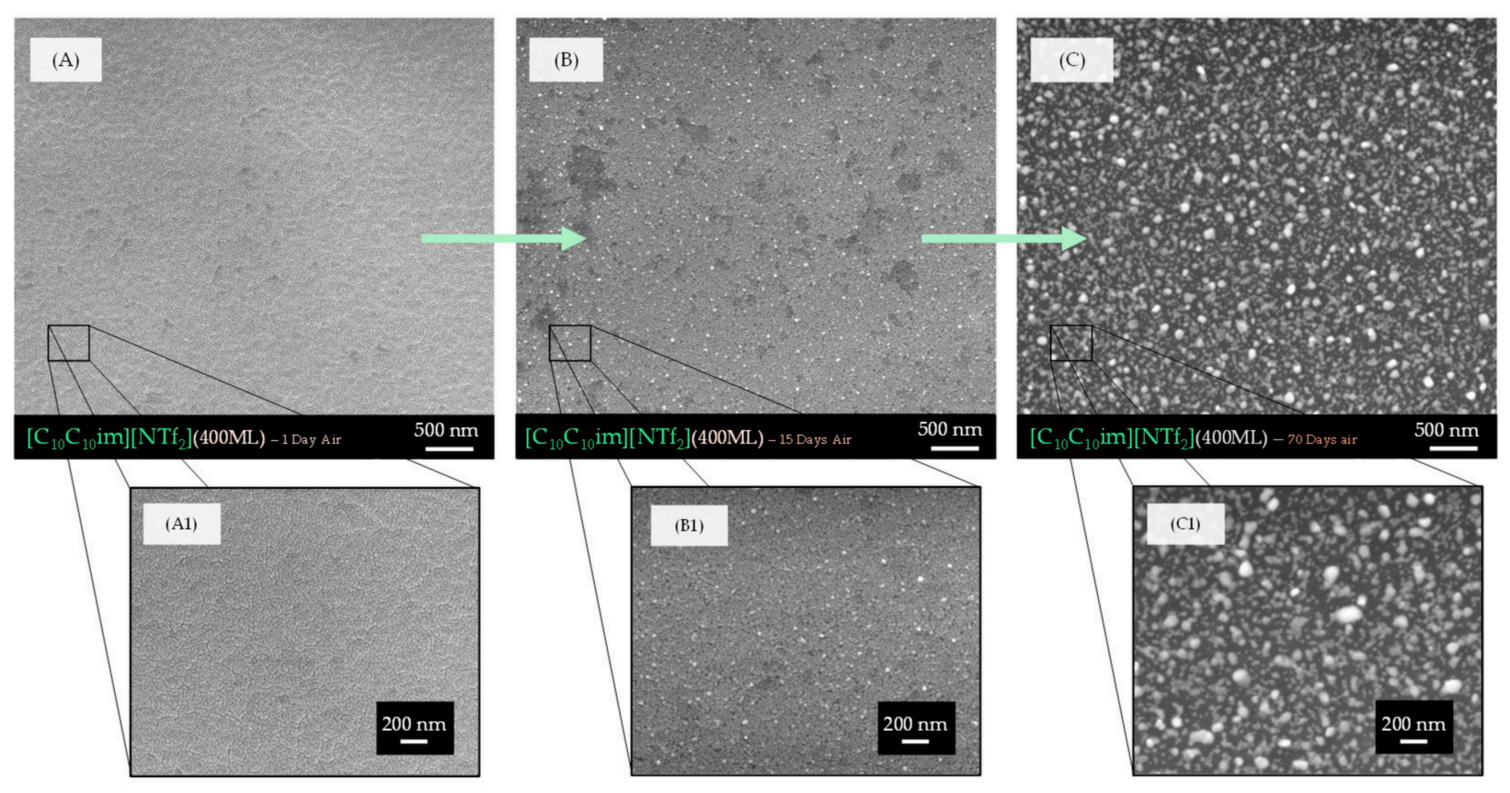
Time-dependent evolution of the surface morphology of a [C_10_C_10_im][NTf_2_] IL film (400 ML) after Ag sputtering (40 s at a discharge current of 20 mA) and subsequent exposure to air. Surface morphology after 1 day of air exposure (**A**) and corresponding close-up (**A1**); after 15 days (**B**) and corresponding close-up (**B1**); and after 70 days (**C**) and corresponding close-up (**C1**). The arrows indicate the increasing exposure time to air. SEM images correspond to top-view micrographs acquired in secondary electron (SE) mode.

The morphology of [C_10_C_10_im][NTf_2_] films before and after Ag sputtering, for both film thicknesses (400 and 600 ML), is shown in Figure 8. Unlike the previous SEM images, which focused on smaller surface regions to analyze AgNPs, these micrographs provide a broader view of the film surface. This larger field of view allows for evaluation of the overall organization of the IL film and assessment of whether the initial droplet-based morphology is preserved after silver deposition. The pre-sputtering images show both films composed of well-defined droplets, with regions enriched in ionic liquid separated by brighter interdomain areas, most likely corresponding to the exposed ITO/glass substrate. After 40 s of Ag sputtering, the surface morphology changed; however, the original droplet-like organization remained clearly identifiable. For both thicknesses, the post-sputtering surfaces consist of darker rounded domains separated by brighter boundaries, resembling the initial droplet distribution observed before sputtering. The 400 ML film exhibited a more regular, well-defined, patterned morphology after sputtering, whereas the 600 ML film appeared more heterogeneous. After 20 days of air exposure, bright particles became visible on the surface. These features are consistent with the particles previously observed at higher magnification in Figure 4. To gain further insight into the aging process induced by air exposure, a complementary time-dependent SEM study was performed using 400 ML [C_10_C_10_im][NTf_2_] films (containing Ag). The samples were analyzed after 1, 15, and 70 days of exposure to air (Figure 9A–C), revealing clear morphological changes over time. One day after Ag deposition, the surface appeared covered by a thin silver film. This morphology suggests that Ag was initially distributed as very small NPs and/or nanosized domains spread across the IL surface. After 15 days of air exposure, numerous particles became visible throughout the film.

Simultaneously, the initially homogeneous silver surface became less continuous, indicating progressive reorganization of the deposited silver. After 70 days, the bright particles became larger and more pronounced, while the initial silver thin film was no longer clearly distinguishable. These observations indicate a gradual aging-induced restructuring process involving nanoparticle growth and/or aggregation at the IL surface. The UV–Vis spectra followed a similar trend. Freshly prepared samples exhibited the lowest SPR band intensity, whereas aged samples showed a progressively stronger optical response. This behavior suggests that the silver species became increasingly organized into plasmonically active nanoparticles during aging, consistent with the morphological evolution observed by SEM.

**Figure 10:**
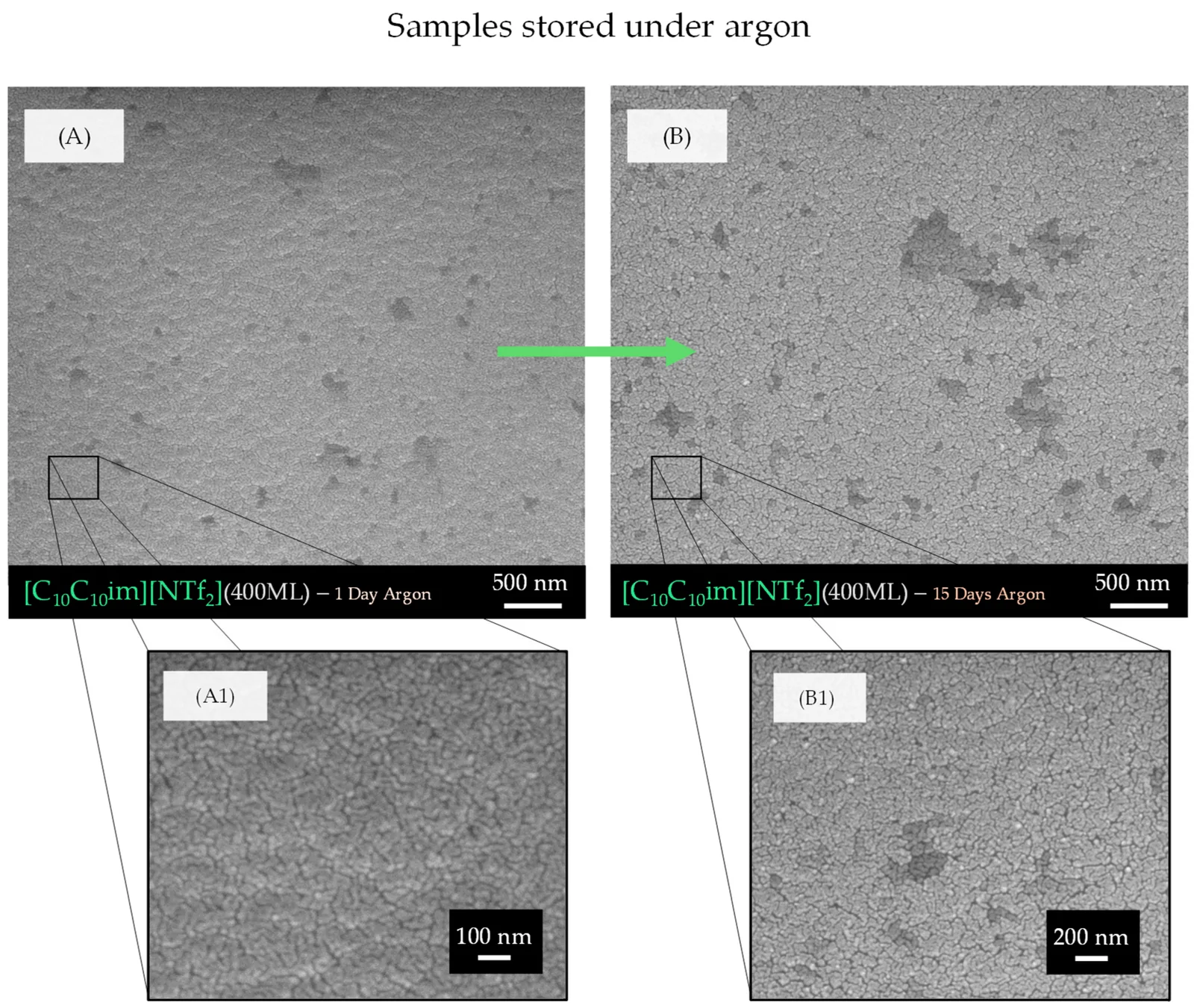
Time-dependent evolution of the surface morphology of a [C_10_C_10_im][NTf_2_] IL film (400 ML) after Ag sputtering (40 s at a discharge current of 20 mA). Surface morphology after 1 day of storage under argon (**A**) and corresponding close-up (**A1**); and after 15 days of storage under argon (**B**) and corresponding close-up (**B1**). The arrow indicates the increasing exposure time to air. SEM images correspond to top-view micrographs acquired in secondary electron (SE) mode.

To further investigate the origin of the bright particles observed after aging, additional [C_10_C_10_im][NTf_2_] films (400 ML) were prepared under identical conditions and subjected to 40 s of Ag sputtering. Instead of being exposed to air, these samples were stored under an argon atmosphere. The sample analyzed after 1 day of storage (Figure 10A) exhibited the formation of a thin Ag film, similar to that observed in the corresponding air-exposed sample. However, after 15 days of storage under argon (Figure 10B), the number of bright particles visible on the surface was substantially lower than that observed for the equivalent sample aged in air. Furthermore, the silver-rich surface layer appeared to be better preserved under argon. These observations indicate that air exposure plays an important role in the aging process of Ag-containing IL films. The markedly lower density of bright particles observed under argon suggests that atmospheric species contribute to the morphological evolution of the deposited silver over time. In particular, oxygen and moisture may promote nanoparticle growth, aggregation, or surface reorganization, leading to the progressive formation of the bright features observed in air-exposed samples. To further highlight the role of the IL in controlling silver organization, a reference experiment was performed by depositing Ag under identical sputtering conditions directly onto bare ITO/glass substrates. The corresponding SEM images are presented in Figures S4–S6. In contrast to the IL-containing systems, rapid silver aggregation was observed immediately after deposition, with the absence of the characteristic plasmonic coloration typically associated with well-dispersed AgNPs. This behavior suggests that, in the absence of the ionic liquid matrix, silver species are not effectively stabilized or guided into controlled growth pathways, leading instead to fast coalescence. These results further support the key role of the IL film in mediating the dispersion, confinement, and time-dependent evolution of Ag nanostructures.

**Figure 11:**
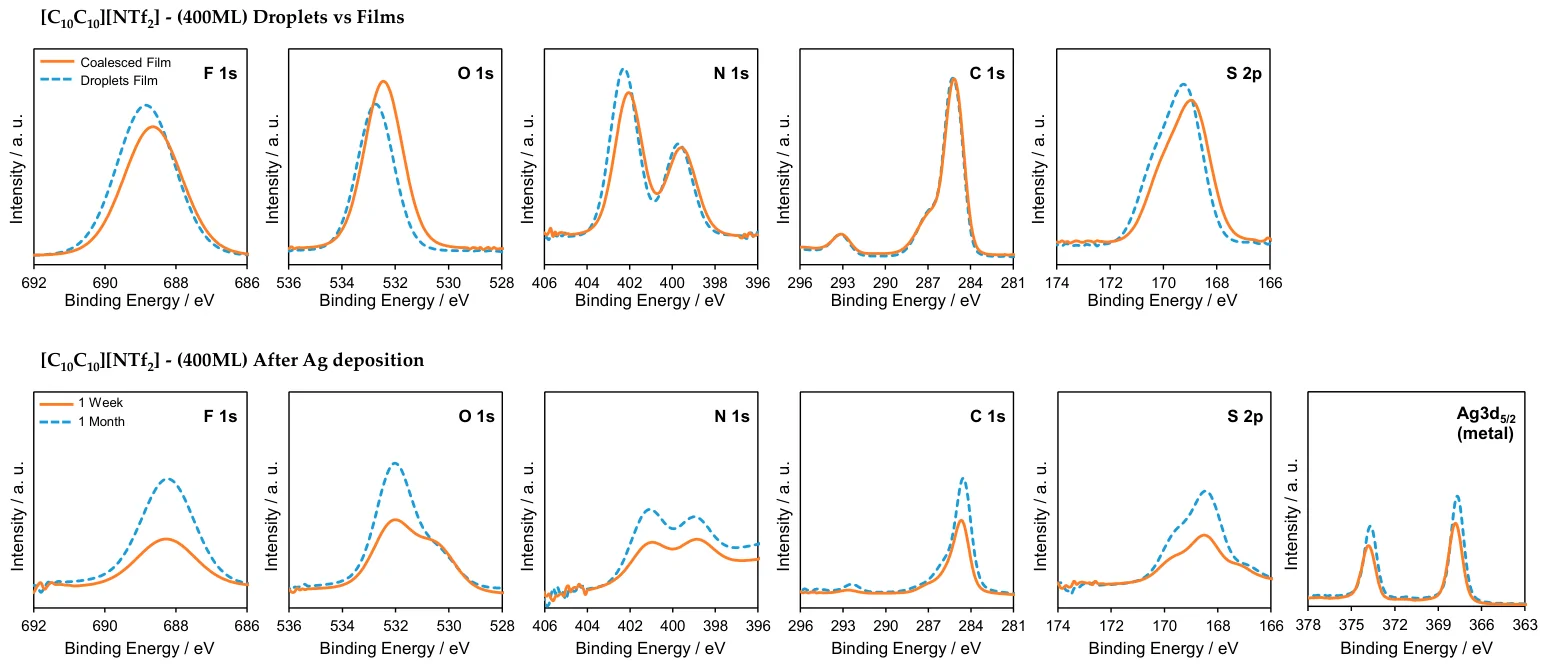
XPS spectra of [C_10_C_10_im][NTf_2_] films (400 ML) exhibiting droplet morphology and a fully coalesced film structure (**top**). XPS spectra of [C_10_C_10_im][NTf_2_] films (400 ML) after Ag sputtering, followed by air exposure for 1 week and 1 month (**bottom**).

To better evaluate the surface composition of [C_10_C_10_im][NTf_2_] films and the effect of plasma/sputtering treatment on the IL surface, XPS analysis was performed, as shown in Figure 11. XPS analysis focused on the 400 ML films because these samples were selected as representative systems for investigating the surface chemical state of Ag and the IL, rather than establishing thickness-dependent trends. Comprehensive XPS results for the bare substrates, IL films, and IL films containing AgNPs, including survey spectra, are presented in Figures S7–S14. In the first experiment, a [C_10_C_10_im][NTf_2_] film composed of micro- and nanodroplets (as-deposited by thermal evaporation) was compared with a coalesced film of identical thickness (400 ML). The coalesced film was obtained after argon plasma treatment without a Ag target, using the same plasma exposure time as in the Ag sputtering experiments. The comparison between the droplet-based and plasma-treated coalesced films revealed only minor differences in the F 1s, O 1s, N 1s, C 1s, and S 2p regions. The high-resolution XPS spectra of the pristine IL droplets exhibit the characteristic binding energies of the IL. The F 1s, O 1s, and S 2p core levels are centered at 688.9, 532.7, and 169.1 eV, respectively. The N 1s spectrum displays two components centered at 402.3 eV (cation) and 399.7 eV (anion). The C 1s spectrum was fitted with four peaks centered at 284.8, 285.5, 286.8, and 293.1 eV, where the first three components originate from the different carbon environments of the cation, and the latter corresponds to the anion. Following plasma treatment, only minor binding-energy shifts (<0.3 eV) are observed for the F 1s, O 1s, N 1s, and S 2p core levels, whereas the C 1s spectrum remains essentially unchanged. These results indicate that the plasma treatment promotes a morphological transition from discrete droplets to a coalesced film without significantly altering the chemical structure of the IL.

In the second experiment, XPS spectra were collected for the [C_10_C_10_im][NTf_2_] film (400 ML) after Ag deposition, followed by exposure to air for 1 week and 1 month. After 1 week, the relative intensity of IL-related signals decreased, consistent with partial coverage of the IL surface by Ag-containing species. Compared with the pristine IL samples, all core levels exhibit systematic shifts towards lower binding energies of more than 0.5 eV, with the largest shift (approximately 1.2 eV) observed for the cationic N 1s component. The surface carbon contribution decreased from approximately 60% in the pristine IL film to approximately 40% after Ag sputtering. In addition, the contributions associated with nitrogen species became less resolved and changed in relative intensity. The cation-to-anion N peak-area ratio decreases relative to the pristine IL, which may indicate preferential interactions between Ag species and specific ionic moieties. The O 1s spectrum develops an additional low-binding-energy component centered at approximately 530.4 eV, while the S 2p spectrum exhibits an additional shoulder after Ag deposition, indicating the coexistence of different sulfur chemical environments. These spectral changes are consistent with modifications of the local chemical environment induced by Ag deposition. The additional O 1s component may also indicate partial surface oxidation of Ag upon exposure to air. The Ag 3d spectra alone do not allow an unambiguous distinction between metallic Ag and oxidized Ag species because of the very small chemical shift between these states. A comparison between the samples aged for one week and one month reveals no significant changes in the binding energies of the analyzed core levels, with only variations in peak intensities being observed. However, after 1 month of air exposure, the IL-related signals increased again, suggesting partial re-exposure of the IL surface, likely associated with reorganization of the NPs initially present on the surface.

## 3. Discussion

The results presented in this work show that the formation and stabilization of AgNPs in imidazolium-based IL films are strongly influenced by several factors, including the alkyl side-chain length of the cation, the IL film morphology, film thickness, and the aging environment. Under constant Ag sputtering conditions, significant differences were observed between [C_7_C_7_im][NTf_2_], [C_8_C_8_im][NTf_2_], and [C_10_C_10_im][NTf_2_], indicating that the choice of IL has a major impact on the organization, mobility, and evolution of the deposited Ag species [9,23]. SEM analysis of the IL films prior to Ag deposition showed that all investigated systems form relatively large and heterogeneous droplets on the ITO/glass surface. This behavior is attributed to the increasing contribution of non-polar domains compared to shorter-chain ILs, which influences wetting behavior, surface mobility, and the balance between IL–IL, IL–ITO, and IL–vacuum interactions [19,20,21,22,23]. The formation of larger droplets reduces the fraction of exposed substrate area. This is particularly relevant for the sputtering process, as a lower exposed ITO surface decreases the probability of direct metal deposition onto the substrate during the initial stages of growth. As a consequence, a larger fraction of incoming silver species interacts with the IL phase, where they can be captured, confined, and stabilized. These observations are consistent with our previous work on shorter-chain imidazolium ILs [9]. In addition, increasing the IL film thickness also promotes droplet growth and surface coverage. However, the combined effect of film thickness and alkyl chain length must be carefully considered to optimize AgNP formation and stabilization. While excessively thick films may tend toward more coalesced morphologies, longer alkyl side chains favor droplet-based structures that enhance nanoparticle confinement. Notably, [C_10_C_10_im][NTf_2_] films with 400 ML thickness already exhibit larger droplets than those previously observed in thicker films of [C_5_C_5_im][NTf_2_]. Overall, these results demonstrate that increasing the alkyl side-chain length promotes a droplet-based morphology that is more effective for Ag capture and nanoparticle stabilization.

Analyzing the SEM images of the freshly sputtered IL films, a large number of well-defined, isolated nanoparticles was not immediately observed on the surface. Instead, the samples appeared to be covered by a very thin silver film distributed across the IL surface. This observation is consistent with the UV–Vis spectra recorded immediately after preparation, where the SPR bands generally exhibited lower intensity. The time-dependent studies were essential to elucidate the morphological and optical evolution of the different samples. After several days of aging, the appearance of brighter and more clearly distinguishable particles was observed on the IL surface. Simultaneously, the UV–Vis spectra showed an increase in the intensity of the SPR band. This correlation between both techniques suggests that aging promotes the transformation of the initially deposited silver layer into more optically active and well-defined nanoparticles. The exact mechanism underlying this transformation is still not fully understood. However, it may involve migration of Ag atoms or small clusters within the IL matrix, as well as fragmentation or reorganization of the initially deposited silver layer, leading to the formation of more isolated nanoparticles. Another possible explanation is the gradual exposure of nanoparticles that were initially embedded in, or partially obscured by, the IL film.

Focusing on the UV–Vis spectra, additional evidence is obtained for the effect of IL alkyl side-chain length on AgNP formation and stability. All samples exhibited absorption bands in the wavelength region characteristic of the SPR of AgNPs, confirming the formation of optically active nanoparticles within the IL films. However, the intensity, width, and position of these bands varied significantly among the different ILs. The [C_7_C_7_im][NTf_2_] films showed lower absorbance values accompanied by more pronounced band broadening and red-shifting upon aging. These spectral features are typically associated with larger particle sizes, broader size distributions, stronger interparticle interactions, or nanoparticle aggregation [60]. Accordingly, although AgNP formation occurs in this system, the IL appears less effective in stabilizing the nanoparticles and preventing growth or aggregation over time. The [C_8_C_8_im][NTf_2_] films exhibited an intermediate behavior. Their SPR bands were generally more intense and less broadened than those of [C_7_C_7_im][NTf_2_], and the position of the maximum absorbance remained closer to that observed for [C_10_C_10_im][NTf_2_]. This indicates that increasing the alkyl side-chain length from C7 to C8 already enhances the stabilization of AgNPs. Nevertheless, the reduced absorbance intensity and residual spectral broadening suggest that this system remains less effective than [C_10_C_10_im][NTf_2_]. Overall, the [C_10_C_10_im][NTf_2_] films displayed the most intense and well-defined SPR bands, with less pronounced red-shifting and broadening. This behavior suggests the formation of a more stable nanoparticle population with reduced aggregation tendency. These results are fully consistent with the SEM observations, where [C_10_C_10_im][NTf_2_] films exhibited a more homogeneous AgNP distribution after aging.

Looking at the film thickness, increasing from 400 ML to 600 ML led to an overall enhancement of the SPR band intensity, particularly for the C7 and C8 systems. This indicates that thicker IL films provide a more favorable environment for AgNP formation. However, the presence of an absorption shoulder becomes more pronounced in these samples, suggesting that increased thickness may also promote the formation or persistence of larger Ag-containing aggregates alongside the nanoparticles. For the C10 system, the differences between 400 and 600 ML films were less significant, likely because even the thinner film already consists of large droplets capable of efficiently capturing and stabilizing Ag species. Nevertheless, the 600 ML film exhibited a more stable optical response over time, pointing to improved long-term stabilization. Overall, increasing IL film thickness enhances AgNP formation and stability up to a certain extent, although it may also lead to a broader distribution of Ag species, including larger particles and aggregates.

The broader SEM images of [C_10_C_10_im][NTf_2_] films before and after silver sputtering provide an additional perspective on the film organization. Unlike the higher-magnification images used for nanoparticle analysis, these micrographs cover a larger surface area and reveal that the original droplet-based morphology is not completely removed by sputtering. After Ag deposition, the surface still exhibits a patterned structure resembling the initial IL droplet distribution. This indicates that the Ag layer is formed on a heterogeneous IL template rather than on a fully coalesced IL film. This morphological “memory” is important for understanding the AgNP formation mechanism. The darker domains observed after sputtering are likely associated with regions containing higher IL content, whereas the brighter boundaries may correspond to thinner IL regions, partially exposed ITO, and/or areas with higher Ag coverage. The persistence of this pattern after sputtering suggests that the IL droplets do not fully merge into a continuous homogeneous layer. Instead, the pre-existing IL morphology continues to govern the spatial distribution, accumulation, and stabilization of the deposited silver species. Overall, these observations support the idea that AgNP formation and organization are governed by a combination of sputtering parameters and the intrinsic mesoscale structure of the IL films.

To further clarify the process of AgNP organization on the surface, additional aging experiments were performed using [C_10_C_10_im][NTf_2_]. After 1 day of air exposure, the surface consistently exhibited the formation of a thin Ag film. After 15 days, small bright particles became visible across the surface, while the initial silver layer appeared less continuous, showing signs of fragmentation. After 70 days, the particles became larger and more clearly defined, and the initial continuous layer was no longer clearly observed. This time evolution suggests that the initially deposited Ag layer progressively reorganizes into more discrete surface nanoparticles. At shorter aging times, this reorganization appears to enhance the optical response by increasing the population of well-defined AgNPs. However, beyond a certain point, further aging leads to particle growth, aggregation, and/or oxidation-assisted transformations, which may broaden the size distribution and reduce optical quality. The possibility of oxidation-assisted processes provides a plausible explanation for the observed evolution. The comparison between air-aged and argon-stored samples supports this interpretation. When stored under argon, the silver-rich layer was more stable, and the number of bright particles after 15 days was significantly lower than in the corresponding air-exposed samples. This indicates that the aging process in air is not solely driven by spontaneous diffusion or IL-mediated reorganization. Instead, exposure to atmospheric species accelerates the morphological transformation of the silver layer. In particular, oxygen and/or moisture are likely to contribute to the observed restructuring of the Ag-containing phase at the IL surface, promoting the formation and growth of discrete nanoparticles over time.

The XPS technique provided complementary information on the surface chemistry of the samples. The quantitative determination of the AgNP layer thickness, surface roughness, and Ag concentration profiles within the XPS information depth cannot be reliably extracted from the available data. Before plasma treatment, the IL forms discrete droplets with heights ranging from several tens to hundreds of nanometers. After plasma treatment, these droplets coalesce into a continuous film, although the nominal film thickness remains above 300 nm (Table S1). It should be noted that the depth distribution of Ag within the IL films may depend on the film thickness due to the diffusion of Ag species after deposition. In the present work, identical deposition conditions were intentionally employed for all samples to ensure that the same amount of Ag was deposited, thereby enabling a direct comparison of the influence of IL film thickness on AgNP formation and evolution. Since the XPS information depth is limited to approximately 10 nm, the local Ag concentration within this near-surface region cannot be determined from the present measurements. Following deposition, Ag species may diffuse within the IL film before nucleating and aggregating into discontinuous nanoparticles. Consequently, photoelectrons originating from both the Ag nanostructures and the underlying IL can be detected within the XPS sampling depth. A more detailed assessment of the Ag depth distribution would require dedicated depth-resolved surface analysis techniques. Advanced approaches, such as those proposed by Gokturk and Suzer for studying IL films under dynamic conditions, may represent a promising direction for future investigations of AgNP/IL systems [61].

The comparison between the droplet-based [C_10_C_10_im][NTf_2_] film and the argon plasma-treated coalesced film revealed only minor differences in the F 1s, O 1s, N 1s, C 1s, and S 2p regions. This is an important observation, as Ag sputtering inherently involves plasma exposure, allowing the effects of plasma treatment on the IL surface to be decoupled from those associated with Ag deposition. The XPS results indicate that the chemical signature of the IL is largely preserved after plasma treatment in the absence of a silver target, suggesting minimal plasma-induced degradation under the conditions employed. For instance, the cation-to-anion N 1s peak-area ratio remained essentially unchanged after plasma treatment (approximately 1.8 before and after plasma treatment), in good agreement with the theoretical stoichiometric value of 2 expected for [C_10_C_10_im][NTf_2_]. Likewise, the F/S ratio remained nearly constant (approximately 3.4 before and after plasma treatment), close to the theoretical value of 3. Together with the very small binding-energy shifts (<0.3 eV), these observations indicate that the argon plasma treatment does not significantly affect the surface stoichiometry or chemical environment of the IL. More pronounced changes were observed after Ag deposition. These can be mainly attributed to the presence of Ag/AgOx species (AgNPs and/or a thin Ag layer), leading to Ag–IL interfacial interactions, as well as subsequent aging effects. After Ag sputtering, the XPS spectra show a decrease and modification in the intensity of IL-related signals. This is primarily attributed to attenuation of photoelectrons from the underlying IL due to partial coverage of the surface by Ag-containing species. Given the high surface sensitivity of XPS, even discontinuous Ag layers or dispersed Ag/AgO_x_ nanoparticles can significantly reduce the detected intensity of the C 1s, N 1s, F 1s, O 1s, and S 2p signals. Accordingly, the decrease in carbon contribution after Ag deposition is consistent with partial surface coverage rather than with chemical degradation of the IL. The N 1s region, which includes contributions from both the imidazolium cation and the [NTf_2_] anion, also changes in relative intensity and spectral shape after Ag deposition, indicating modifications in the surface chemical environment. These changes may arise from several concurrent processes, including possible Ag–cation interactions, IL surface reorganization associated with droplet coalescence and nanoparticle formation, local charging effects, and interactions between Ag/AgO_x_ species and the anion, particularly via the oxygen-containing sulfonyl groups. Overall, the N 1s evolution suggests that Ag deposition affects both the surface organization and the local chemical environment of the ionic liquid. Before Ag deposition, the N 1s region was fitted with two well-defined components centered at approximately 402.0 eV and 399.5 eV. The higher binding energy component at ~402.0 eV is the most intense and is attributed to the imidazolium cation, whereas the lower binding energy component at ~399.5 eV is assigned to the [NTf_2_]^−^ anion. The higher intensity of the N 1s component associated with the imidazolium cation is expected, at least in part, based on stoichiometric considerations (two nitrogen atoms in the cation versus one in the [NTf_2_]^−^ anion), although additional contributions such as surface composition and interfacial organization of the IL film may also play a role. After Ag deposition, both N 1s components decrease in intensity and become less distinct, indicating a reduction in the spectral contrast between the two ionic species at the outermost surface. In addition, a shift in the components is observed, with the cation-related peak moving to ~401.0 eV and the anion-related peak remaining close to ~399.0 eV. As a consequence, the separation between the two contributions decreases. These changes are consistent with a modification of the local electrostatic environment and surface potential induced by the presence of Ag species and the associated reorganization of the IL at the interface. Such behavior may arise from a combination of interfacial restructuring of the IL induced by Ag deposition and nanoparticle formation, electrostatic screening effects caused by Ag/AgOx species at the surface, and redistribution of the ionic components within the near-surface region. However, based on XPS alone, it is not possible to assign these changes to specific chemical bonding between Ag and either ionic species. The Ag 3d region confirms the presence of silver at the surface. An increase in Ag 3d intensity upon aging indicates greater surface exposure or redistribution of silver species toward the outermost surface over time. This observation is consistent with the SEM results, which show an increased density of bright particles after aging, and with the UV–Vis data, where the SPR intensity increases during the initial aging period. However, the precise distinction between metallic Ag and oxidized silver species (Ag_2_O, or AgO) cannot be unambiguously established from the Ag 3d region alone, due to the close proximity of their binding energies. When considered together with the evolution of the O 1s and Ag 3d signals, the formation of partially oxidized silver species at the surface becomes a plausible scenario.

Considering all results, a consistent picture of the system can be proposed. During sputtering, silver is deposited onto a droplet-structured [C_10_C_10_im][NTf_2_] film, initially forming a thin Ag film that partially reflects the underlying IL morphology. At this stage, silver is not predominantly present as well-defined, optically efficient nanoparticles, which is consistent with the relatively weak initial SPR response. During aging, particularly under air exposure, the silver layer undergoes migration, reorganization, and partial transformation, leading to the emergence of more distinct nanoparticles observed by SEM and a concomitant increase in SPR intensity in UV–Vis spectra. In contrast, storage under argon significantly suppresses this evolution, indicating that the system is kinetically stabilized in the absence of atmospheric species. Importantly, exposure to air does not imply the formation of AgNPs from scratch, since silver is already present immediately after sputtering. Instead, atmospheric exposure provides a dynamic environment that promotes the diffusion, reorganization, and partial oxidation of pre-existing Ag species, enhancing their transformation into more optically active and morphologically distinguishable NPs. At longer aging times, excessive oxidation and aggregation processes may also contribute to changes in particle size distribution and optical response. XPS analysis supports this interpretation by evidencing surface coverage effects, preservation of the IL chemical framework after plasma treatment, and progressive changes in the chemical environment of Ag-containing species at the surface, consistent with their redistribution and partial oxidation during aging.

Hence, the present study highlights the strong influence of ionic liquid molecular structure and interfacial organization on the formation and time-dependent evolution of Ag nanostructures. The ability to tune nanoparticle stabilization and optical response through the alkyl side-chain length and film thickness demonstrates the potential of ionic liquid thin films as adaptable templates for metal nanoparticle growth. Moreover, the time-dependent reorganization observed under ambient conditions provides further insight into how external environmental factors can be used to modulate nanoparticle morphology and optical properties. Since the ILs employed in this work can be thermally evaporated under high-vacuum conditions, their removal after AgNP formation may offer a potential route for isolating the nanoparticles. However, the stability and integrity of the resulting nanostructures following IL removal should be evaluated in future studies. These findings may contribute to the rational design of ionic liquid-based platforms for controlled nanoparticle synthesis, plasmonic systems, and functional surfaces.

## 4. Materials and Methods

### 4.1. Ionic Liquids

The ILs 1,3-diheptylimidazolium bis(trifluoromethanesulfonyl)imide ([C_7_C_7_im][NTf_2_]), 1,3-dioctylimidazolium bis(trifluoromethanesulfonyl)imide ([C_8_C_8_im][NTf_2_]) and 1,3-didecylimidazolium bis(trifluoromethanesulfonyl)imide ([C_10_C_10_im][NTf_2_]) were purchased from IoLiTec (Heilbronn, Germany) with a stated purity of >99%. Prior to film deposition, all ILs were subjected to vacuum thermal evaporation (*p* < 10^−4^ Pa, 473 K) for 30 min to remove volatile content. The IL film deposition was performed only after this pre-conditioning step.

### 4.2. Vacuum Thermal Evaporation of Ionic Liquids

The deposition of micro and nanodroplets of [C_7_C_7_im][NTf_2_], [C_8_C_8_im][NTf_2_], and [C_10_C_10_im][NTf_2_] was performed on indium tin oxide (ITO)-coated glass substrates (~180 nm thickness). The ITO/glass substrates (10 mm × 10 mm × 1.1 mm) were purchased from Praezisions Glas & Optik GmbH (Iserlohn, Germany). IL films were deposited using a customized physical vapor deposition setup based on the Knudsen effusion method (ThinFilmVD), as previously described in the literature [19,20,21,22,23,62] (Figures S15–S17). This approach allows precise control of the mass flow rate (*Φ*) through Knudsen effusion cells. The flux reaching the substrate surface (*Φ*_s_) can be related to the flux at the Knudsen cell orifice (*Φ*_kc_) through a geometric factor (*g*), which depends on the source–substrate distance and deposition geometry. A 6 MHz gold-coated quartz crystal microbalance (QCM) was used to monitor and control *Φ*_s_ in real time. *Φ*_kc_ is described by Equation (1), where *T* is the effusion temperature, *p* is the vapor pressure at *T*, *w*_o_ is the transmission probability factor, *m* is the mass of the effusing species, *M* is the molar mass, *t* is the deposition time, and *A*_o_ is the area of the Knudsen cell orifice [62].

(1) $$\Phi_{s}=g\;\cdot \;\Phi_{\mathrm{kc}}=g\cdot \frac{p\;\cdot \;w_{o}\cdot \sqrt{M}}{\sqrt{2\pi \mathrm{RT}}}=g\cdot \frac{m}{A_{o}\cdot t}$$

The deposition experiments were carried out under high-vacuum conditions achieved through the combined use of a rotary pump and a diffusion pump, allowing the chamber pressure to be reduced to below 10^−4^ Pa. The ITO/glass substrates were maintained at a constant temperature of *T* = 283 K. The experimental conditions used for film fabrication were held constant across all samples, with mass flow rates at the substrate surface (*Φ*_s_) ranging from 0.08 to 0.11 nm s^−1^. The evaporation temperatures for each IL were as follows: *T* ≈ 498.2 K for [C_7_C_7_im][NTf_2_] and [C_8_C_8_im][NTf_2_], and *T* ≈ 513.2 K for [C_10_C_10_im][NTf_2_], corresponding to vapor pressures of ≈0.1 Pa [63]. The use of Knudsen effusion cells enables precise control of the deposition rate, resulting in accurate film thickness control and high reproducibility. The thickness of the IL films is expressed in monolayers (ML), as commonly reported for IL films. Since the deposited material forms micro- and nanodroplet structures rather than a continuous homogeneous layer, the ML unit provides a more meaningful description of the nominal amount of deposited material than a physical thickness expressed in nanometers. 1 ML is defined as a nominal close-packed layer of ion pairs, and its thickness can be estimated using Equation (2) [64].

(2) $$h=\sqrt[{3}]{\frac{V_{m}}{N_{A}}}=\sqrt[{3}]{\frac{M}{N_{A}\times \rho }}$$

where *M* is the molar mass of the IL, *V_m_* is the bulk molecular volume, *ρ* the mass density and *N_A_* is the Avogadro constant. Using this equation, the thickness corresponding to 1 ML was estimated as: *h* = 9.1 Å for [C_7_C_7_im][NTf_2_] (*M* = 545.7 g·mol^−1^ and *ρ* = 1.24 g·cm^−3^ at 283.2 K), *h* = 9.2 Å for [C_8_C_8_im][NTf_2_] (*M* = 573.8 g·mol^−1^ and *ρ* = 1.21 g·cm^−3^ at 283.2 K), and *h* = 9.7 Å for [C_10_C_10_im][NTf_2_] (*M* = 629.8 g·mol^−1^ and *ρ* = 1.16 g·cm^−3^ at 283.2 K) [65]. Based on these values, films with nominal thicknesses of 400 ML correspond to approximately 361, 371, and 387 nm for [C_7_C_7_im][NTf_2_], [C_8_C_8_im][NTf_2_], and [C_10_C_10_im][NTf_2_], respectively. Similarly, films with nominal thicknesses of 600 ML correspond to approximately 543, 557, and 581 nm, respectively.

### 4.3. Sputter Deposition of Silver Particles in ILs

The deposition of Ag was carried out by pulsed DC magnetron sputtering using a Cressington 108 Auto Sputter Coater system (Cressington Scientific Instruments Ltd., Watford, UK). A high-purity (>99.9%) Ag target was used. During the sputtering process, an argon plasma is generated at low pressure (<10 Pa), which is sustained throughout the deposition at a discharge current of 20 mA. In addition to metal deposition, the argon plasma induces structural reorganization of the IL films, promoting coalescence of the micro- and nanodroplets and leading to the formation of a more continuous IL layer [22]. The deposition time was fixed at 40 s for all samples. The amount of deposited Ag was monitored using a QCM, corresponding to a nominal Ag loading of 13.6 μg∙cm^−2^. This value corresponds to an estimated equivalent thickness of approximately 13 nm, assuming the formation of a continuous Ag film with uniform density on a flat substrate. Therefore, this thickness should be considered a nominal equivalent value rather than the actual morphology of the deposited Ag layer. The target–substrate distance was maintained at ~3.8 cm.

### 4.4. High-Resolution Scanning Electron Microscopy (SEM)

The morphological characterization of the IL films prior to Ag deposition was performed using a compact scanning electron microscope (SEM, Hitachi FlexSEM 1000, Hitachi High-Tech Corporation, Tokyo, Japan). The images were acquired at a magnification of 900× using a backscattered electron (BSE) detector, with an accelerating voltage of 3 kV. The working distance was maintained between 6 and 9 mm. This configuration allowed visualization of the IL micro- and nanodroplet morphology on the ITO substrate. After Ag deposition, the morphological and topographical analysis was carried out using a high-resolution field-emission scanning electron microscope (FE-SEM, FEI Quanta 400 FEG ESEM/EDAX Genesis X4M, FEI Europe, Eindhoven, The Netherlands), at CEMUP–Centro de Materiais da Universidade do Porto. Secondary electron (SE) imaging was primarily used to analyze surface morphology, providing high-resolution information on surface texture, roughness of the IL droplet structures, and the presence, size, distribution, and possible aggregation of AgNPs after deposition. Backscattered electron (BSE) imaging was also employed to provide compositional contrast, allowing differentiation between IL-rich regions and Ag-containing domains, although at a lower spatial resolution than SE imaging. SEM micrographs were acquired at magnifications of 50,000×, 100,000×, and 200,000×. All samples were prepared either on the day of analysis or the preceding day and stored under desiccated conditions prior to SEM measurements.

### 4.5. UV-Vis Spectroscopy

UV-Vis analysis was performed on the IL films ([C_7_C_7_im][NTf_2_], [C_8_C_8_Im][NTf_2_], and [C_10_C_10_Im][NTf_2_]) containing AgNPs using an Agilent 8453 UV–Vis diode-array spectrophotometer (Agilent Technologies, Santa Clara, CA, USA). Spectra were recorded in the wavelength range of 200–800 nm at 298.2 K. The presence of AgNPs in the IL films was confirmed by the appearance of the characteristic SPR band in the 400–440 nm region, depending on the size, distribution, and local environment of the nanoparticles.

### 4.6. X-Ray Photoelectron Spectroscopy (XPS) Analysis

XPS was used to characterize bare ITO substrates, Ag-deposited ITO substrates, and IL films ([C_8_C_8_im][NTf_2_] and [C_10_C_10_im][NTf_2_]) deposited on ITO, as well as the same IL films after Ag deposition. Measurements were performed using an ESCALAB 250Xi XPS spectrometer (Thermo Scientific, Waltham, MA, USA) equipped with a monochromatic Al Kα X-ray source (*hν* = 1486.6 eV), operated at 200 W. The analysis area was defined by a 650 µm X-ray spot. Photoelectron spectra were acquired at a take-off angle of 90° relative to the sample surface using a hemispherical analyzer operated in constant analyzer energy (CAE) mode. Survey spectra were recorded with a pass energy of 150 eV, while high-resolution spectra were acquired at a pass energy of 40 eV. Energy step sizes of 1 eV and 0.1 eV were used for survey and high-resolution scans, respectively. High-resolution spectra were fitted using a Gaussian–Lorentzian line shape after subtraction of a Shirley-type background. The binding energy scale was referenced to the C 1s peak of adventitious carbon at 284.8 eV to correct for possible surface charging [66,67]. The elemental compositions (of the detected elements are summarized in the Supporting Information. Core levels analyzed included C 1s, O 1s, N 1s, F 1s, S 2p, In 3d, Sn 3d, and Ag 3d. Data processing was carried out using CasaXPS software (version 2.3, Casa Software Ltd., Devon, UK), and relative sensitivity factors were taken from the Kratos Library [68].

## 5. Conclusions

This work demonstrates that the formation, spatial organization, and long-term evolution of AgNPs in imidazolium-based ionic liquid films are strongly governed by the molecular structure of the IL, the resulting mesoscale film morphology, and the external aging environment. Systematic comparison of [C_7_C_7_im][NTf_2_], [C_8_C_8_im][NTf_2_], and [C_10_C_10_im][NTf_2_] films revealed that increasing the alkyl side-chain length promotes the formation of larger and more heterogeneous microdroplet domains on ITO substrates. This structural evolution reduces the exposed substrate area and enhances the confinement of sputtered silver species within the ionic liquid phase, thereby favoring nanoparticle nucleation and stabilization. Among the systems studied, [C_10_C_10_im][NTf_2_] exhibited the most efficient stabilization of AgNPs, producing a more homogeneous spatial distribution and a more stable plasmonic response over time. This behavior arises from the combined effect of stronger non-polar interactions, improved droplet-based templating, and more effective confinement of the deposited metal species. Importantly, the results show that AgNPs formation in these systems is not an instantaneous process but rather a dynamic, time-dependent evolution. Immediately after sputtering, silver is predominantly present as a thin film rather than as fully developed nanoparticles. Subsequent aging drives a progressive restructuring of the Ag domains into more defined, optically active nanoparticles, as confirmed by SEM and UV–Vis spectroscopy. This transformation is significantly accelerated under air, while storage under argon largely suppresses it, highlighting the key role of atmospheric species in governing interfacial dynamics. XPS analysis further supports this trend by confirming partial surface coverage after Ag deposition, preservation of the ionic liquid chemical framework under plasma exposure, and the evolution of Ag-related species consistent with interfacial reorganization and partial oxidation during aging.

Overall, this study highlights ionic liquid thin films as dynamic and structurally tunable soft templates for metal nanoparticle synthesis. Crucially, ionic liquids constitute a broadly adaptable materials platform for in situ nanofabrication. Their intrinsic properties—negligible vapor pressure and, therefore, excellent compatibility with vacuum-based processes, high thermal stability, and a vast chemical design space enabled by independent variation in cation/anion combinations—make them uniquely suited for controlled nanostructure formation under diverse processing conditions. This combination of structural tunability and robust physical stability further reinforces the potential of ILs as a general and powerful platform for in situ nanoparticle fabrication, providing a framework for the rational design of functional plasmonic and nanostructured interfaces.

## Data Availability

The raw data supporting the conclusions of this article will be made available by the authors on request.

## Supplementary Materials

The following supporting information can be downloaded at: https://www.mdpi.com/article/10.3390/molecules31162798/s1, Figure S1: Photographs and representative SEM images of [C_10_C_10_im][NTf_2_] films before and after Ag sputtering; Figure S2: SEM micrographs of [C_7_C_7_im][NTf2] and [C_10_C_10_im][NTf_2_] films deposited on ITO/glass substrates; Figure S3: Illustration of the typical mechanisms of nucleation and growth of ionic liquid micro- and nanodroplets (precursor ionic liquid films); Figure S4: Time-dependent evolution of the surface morphology of bare ITO/glass substrates and ITO/glass substrates coated with a [C_10_C_10_im][NTf2] IL film after Ag sputtering; Figure S5: Detailed morphology of the indium tin oxide (ITO)/glass surface; Figure S6: Micrographs of ITO-coated glass substrates treated with argon plasma and silver particles; Figure S7: XPS survey spectra of the ITO/glass surface (substrate exposed to air); Figure S8: XPS survey spectra of the ITO/glass surface after removal of adventitious carbon by argon sputtering; Figure S9: XPS survey spectra of the Ag/ITO/glass surface (substrate exposed to air); Figure S10: XPS survey spectra of the Ag/ITO/glass surface after removal of adventitious carbon by argon sputtering; Figure S11: XPS survey spectrum of a [C_10_C_10_im][NTf2] film as-deposited by vacuum thermal evaporation (micro- and nanodroplets); Figure S12: XPS survey spectrum of a [C_10_C_10_im][NTf_2_] film after argon plasma treatment (coalesced IL film); Figure S13: XPS survey spectrum of a [C_10_C_10_im][NTf_2_] film after silver sputtering and 1 week of air exposure; Figure S14: XPS survey spectrum of a [C_10_C_10_im][NTf_2_] film after silver sputtering and 1 month of air exposure; Figure S15: Schematic representation of the vapor deposition/vacuum thermal evaporation methodology (Thin Film VD); Figure S16: Schematic representation of the ovens of the Thin Film VD apparatus; Figure S17: Schematic representation and images of the substrate support system; Table S1: Experimental conditions for the physical vapor deposition of each ionic liquid.
