# Supplementary material for: Silver Deposition on Thin Films of Symmetric Long-Chain Dialkylimidazolium-Based Ionic Liquids
Source: Molecules. 2026 Aug 11;31(16):2798. doi: 10.3390/molecules31162798 (PMC13515322; doi:10.3390/molecules31162798)
Supplement: Supplementary file 1 [file molecules-31-02798-s001.zip › molecules-4446460-supplementary.pdf]

Supporting Information

Silver Deposition on Thin Films of Symmetric Long-Chain Dialkylimidazolium-Based Ionic Liquids

Alexandre C. P. M. Alves, Luís M. N. B. F. Santos, and José C. S. Costa *

CIQUP, Institute of Molecular Sciences (IMS), Department of Chemistry and Biochemistry, Faculty of Science, University of Porto, Rua do Campo Alegre s/n, P4169-007 Porto, Portugal

* *Correspondence: jose.costa@fc.up.pt*

Index

Figure S1. Photographs and representative SEM images of [$C_{10}C_{10}$im][NTf_2] films before and after Ag sputtering. **S3**

Figure S2. SEM micrographs of [C_7C_7im][NTf_2] and [$C_{10}C_{10}$im][NTf_2] films deposited on ITO/glass substrates. **S4**

Figure S3. Illustration of the typical mechanisms of nucleation and growth of ionic liquid micro- and nanodroplets (precursor ionic liquid films). **S4**

Figure S4. Time-dependent evolution of the surface morphology of bare ITO/glass substrates and ITO/glass substrates coated with a [$C_{10}C_{10}$im][NTf_2] IL film after Ag sputtering. **S5**

Figure S5. Detailed morphology of the indium tin oxide (ITO)/glass surface. **S6**

Figure S6. Micrographs of ITO-coated glass substrates treated with argon plasma and silver particles. **S6**

Figure S7. XPS survey spectra of the ITO/glass surface (substrate exposed to air). **S7**

Figure S8. XPS survey spectra of the ITO/glass surface after removal of adventitious carbon by argon sputtering. **S7**

Figure S9. XPS survey spectra of the Ag/ITO/glass surface (substrate exposed to air). **S8**

Figure S10. XPS survey spectra of the Ag/ITO/glass surface after removal of adventitious carbon by argon sputtering. **S8**

Figure S11. XPS survey spectrum of a [$C_{10}C_{10}$im][NTf_2] film as-deposited by vacuum thermal evaporation (micro- and nanodroplets). **S9**

Figure S12. XPS survey spectrum of a [$C_{10}C_{10}$im][NTf_2] film after argon plasma treatment (coalesced IL film). **S9**

Figure S13. XPS survey spectrum of a [$C_{10}C_{10}$im][NTf_2] film after silver sputtering and 1 week of air exposure. **S10**

Figure S14. XPS survey spectrum of a [$C_{10}C_{10}$im][NTf_2] film after silver sputtering and 1 month of air exposure. **S10**

Figure S15. Schematic representation of the vapor deposition/vacuum thermal evaporation methodology (Thin Film VD). **S11**

Figure S16. Schematic representation of the ovens of the Thin FilmVD apparatus. **S11**

Figure S17. Schematic representation and images of the substrate support system. **S12**

Table S1. Experimental conditions for the physical vapor deposition of each ionic liquid. **S13**

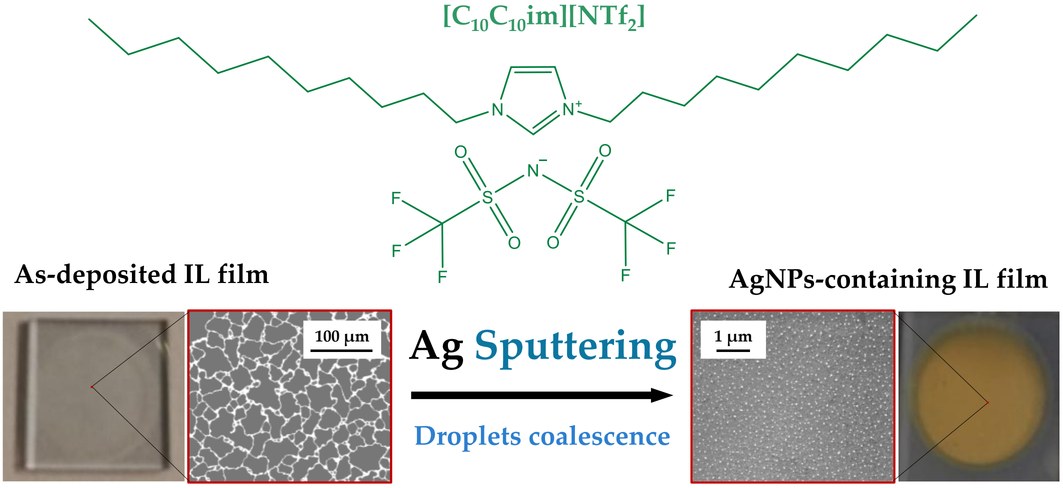

Figure S1. Photographs and representative SEM images of $[C_{10}C_{10}im][NTf_2]$ films before (left) and after Ag sputtering (right). The yellowish coloration is typically associated with the surface plasmon resonance of Ag nanoparticles.

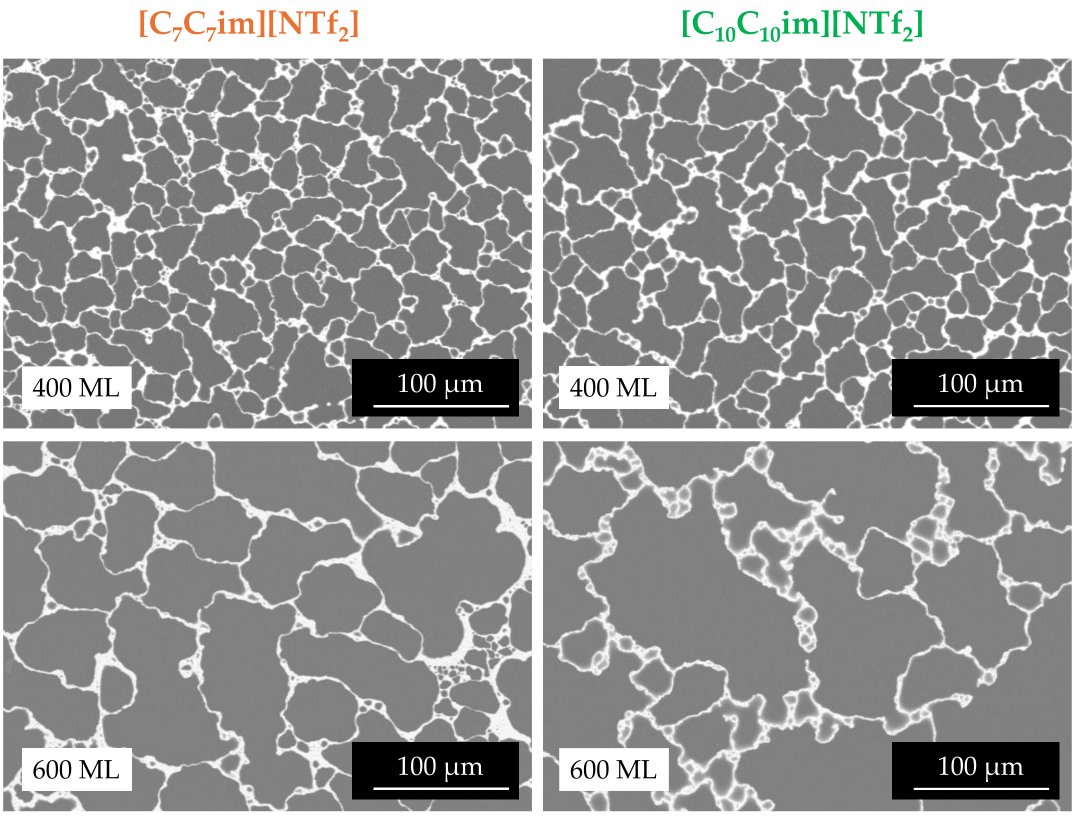

Figure S2. SEM micrographs of [C_7C_7im][NTf_2] (left) and [$C_{10}C_{10}$im][NTf_2] (right) films deposited on ITO/glass substrates. The images correspond to top-view SEM micrographs acquired in backscattered electron (BSE) mode for IL thicknesses of 400 ML (top) and 600 ML (bottom).

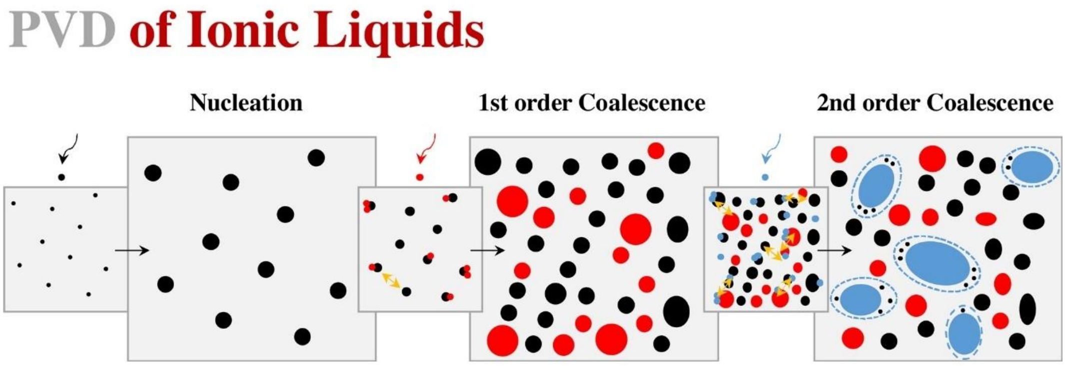

Figure S3. Illustration of the typical mechanisms of nucleation and growth of ionic liquid micro- and nanodroplets (precursor ionic liquid films): minimum free area to promote nucleation (MFAN); first-order coalescence; second-order coalescence. More details: *Appl. Surf. Sci.*, 2018, 428, 242.

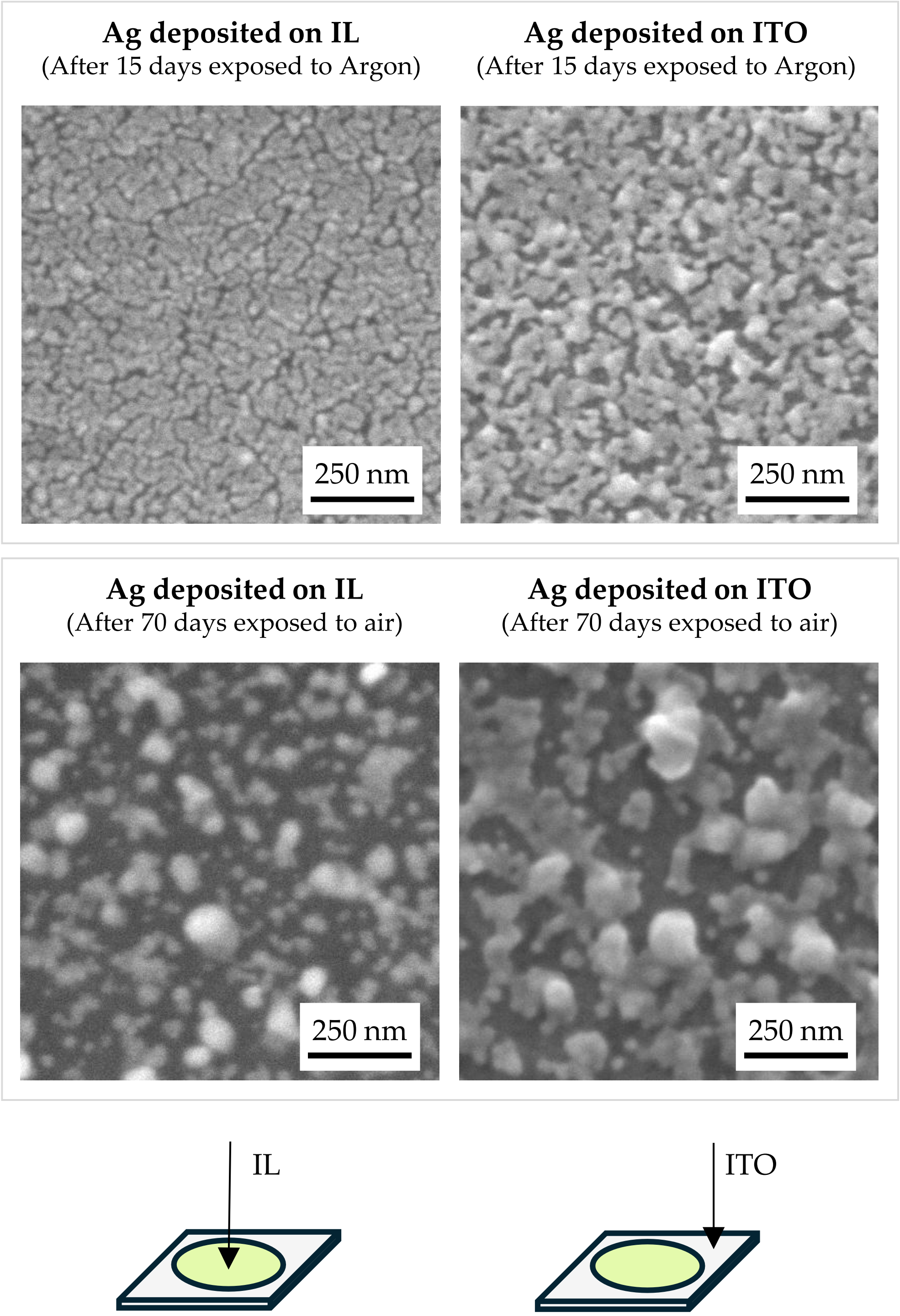

Figure S4. Time-dependent evolution of the surface morphology of bare ITO/glass substrates (right) and ITO/glass substrates coated with a $[C_{10}C_{10}im][NTf_2]$ IL film (400 ML) (left) after Ag sputtering (40 s at a discharge current of 20 mA). The Ag deposited directly onto bare ITO shows immediate and pronounced aggregation. In contrast, Ag deposition onto the IL-coated surface leads to the formation of an initial thin film of AgNPs, which exhibits a characteristic yellowish coloration associated with the surface plasmon resonance of AgNPs. In the absence of the IL, no such coloration is observed. After 15 days of storage under argon (top) and 70 days of exposure to air (bottom), morphological evolution is observed in both systems, with aggregation and oxidation-related effects being significantly more pronounced on the bare ITO surface than on the IL-coated substrate. SEM images correspond to top-view micrographs acquired in secondary electron (SE) mode.

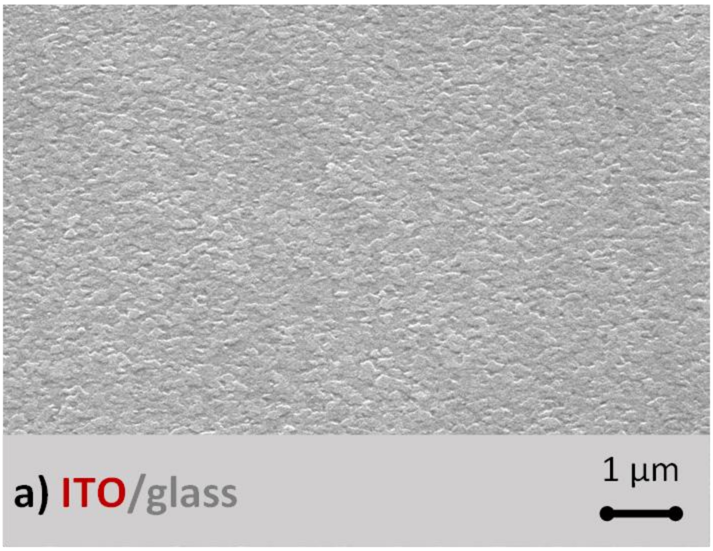

Figure S5. Detailed morphology of the indium tin oxide (ITO)/glass surface. Micrograph acquired through high-resolution scanning electron microscopy (SEM) by using a secondary electron detector (SED). A lateral view at 45° was obtained with a magnification of 25000×.

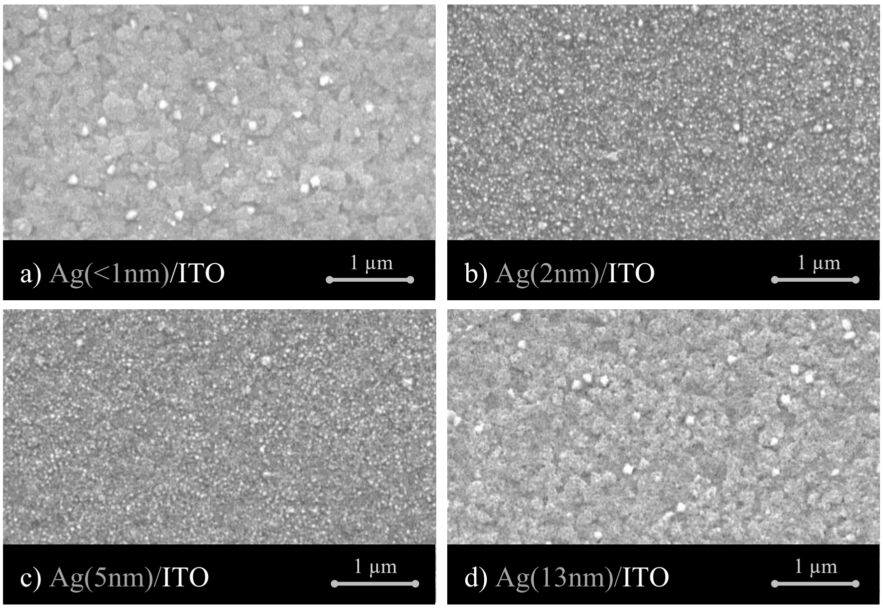

Figure S6. Micrographs of ITO-coated glass substrates treated with argon plasma and silver (Ag) particles. Morphology of the ITO surface after 5 (1 nm, image a), 10 (2 nm, image b), 20 (5 nm, image c), and 40 (13 nm, image d) s of sputter deposition from a silver magnetron target and argon plasma using a discharge current of 20 mA. Top views (50000 ×) were acquired through high-resolution scanning electron microscopy (SEM) by using a secondary electrons detector (SED).

For lower amounts of Ag (2 or 5 nm, images b, and c), the formation of individual Ag clusters onto ITO can be observed. The increase of the Ag thickness (13 nm, image d) leads to the coalescence of the clusters and the formation of a thin film of Ag onto the substrate.

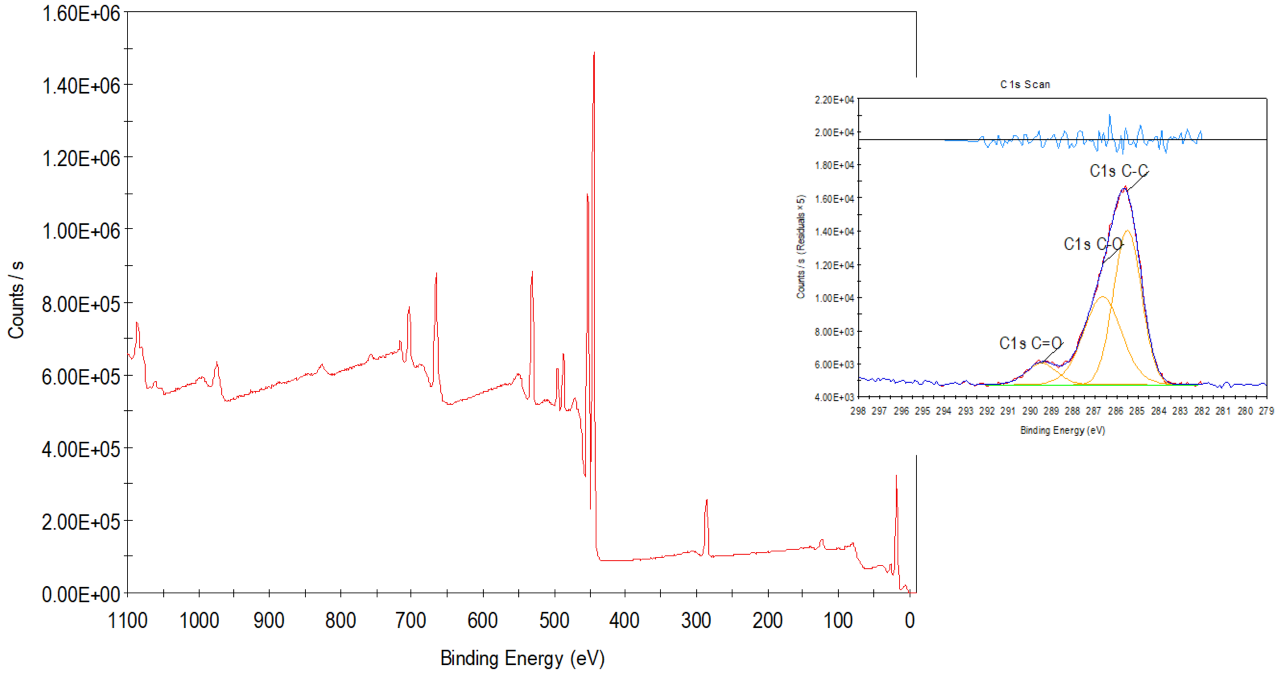

Figure S7. XPS survey spectra of the ITO/glass surface (substrate exposed to air). The In 3d region shows peaks at 444.9 eV (In $3d_{5/2}$) and 452.4 eV (In $3d_{3/2}$), while the Sn 3d region shows peaks at 486.9 eV (Sn $3d_{5/2}$) and 495.3 eV (Sn $3d_{3/2}$), consistent with the presence of indium tin oxide (ITO) at the surface. The C1s spectrum reveals adventitious carbon contamination on the surface, with detected C–C, C–O, and C=O components.

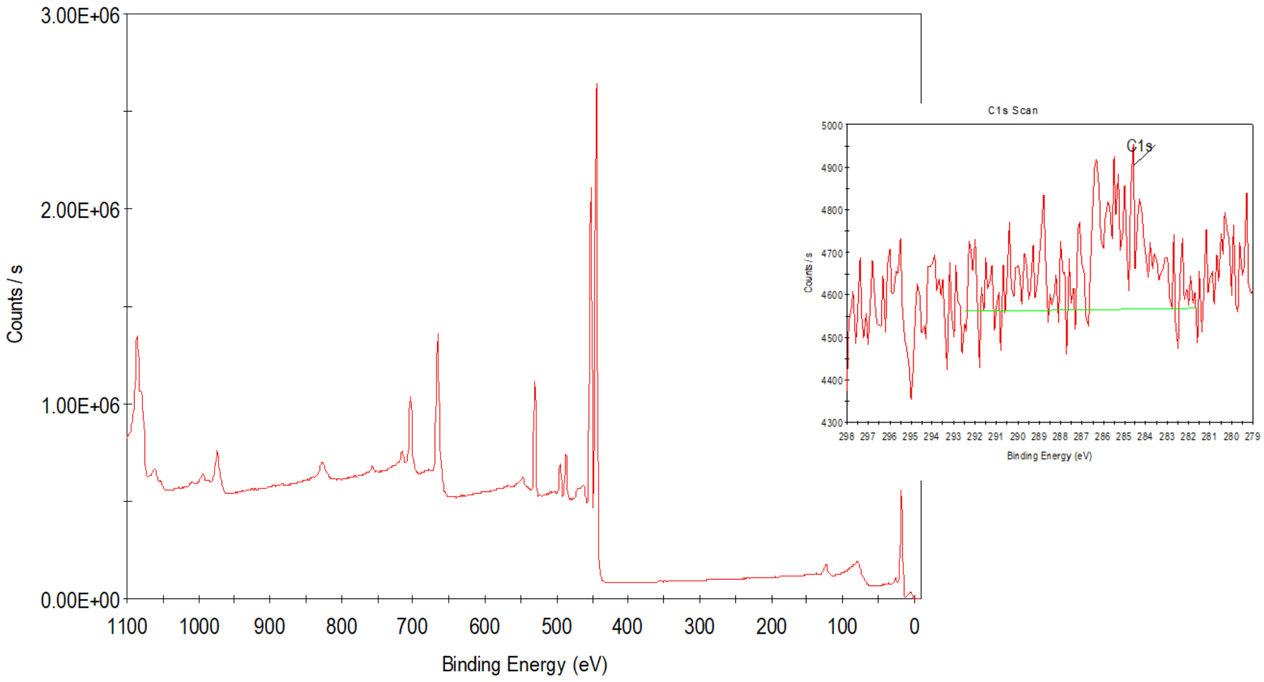

Figure S8. XPS survey spectra of the ITO/glass surface after removal of adventitious carbon by argon sputtering. The In 3d region shows peaks at 444.5 eV (In $3d_{5/2}$) and 452.0 eV (In $3d_{3/2}$), while the Sn 3d region shows peaks at 486.6 eV (Sn $3d_{5/2}$) and 494.9 eV (Sn $3d_{3/2}$), consistent with the presence of indium tin oxide (ITO) at the surface.

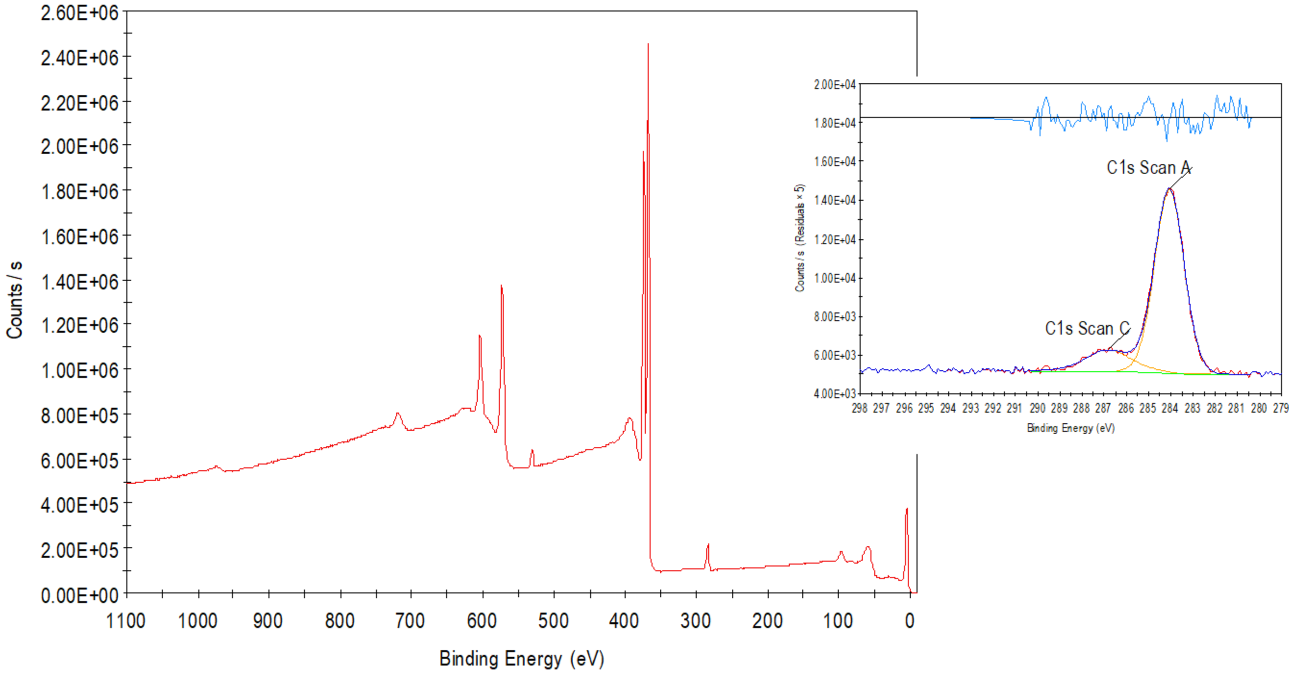

Figure S9. XPS survey spectra of the Ag/ITO/glass surface (substrate exposed to air). The Ag 3d region shows two peaks at 367.8 eV (Ag $3d_5/_2$) and 373.8 eV (Ag $3d_3/_2$), with a spin–orbit splitting of 6.0 eV, consistent with the presence of metallic silver at the surface. The C1s spectrum reveals adventitious carbon contamination on the surface.

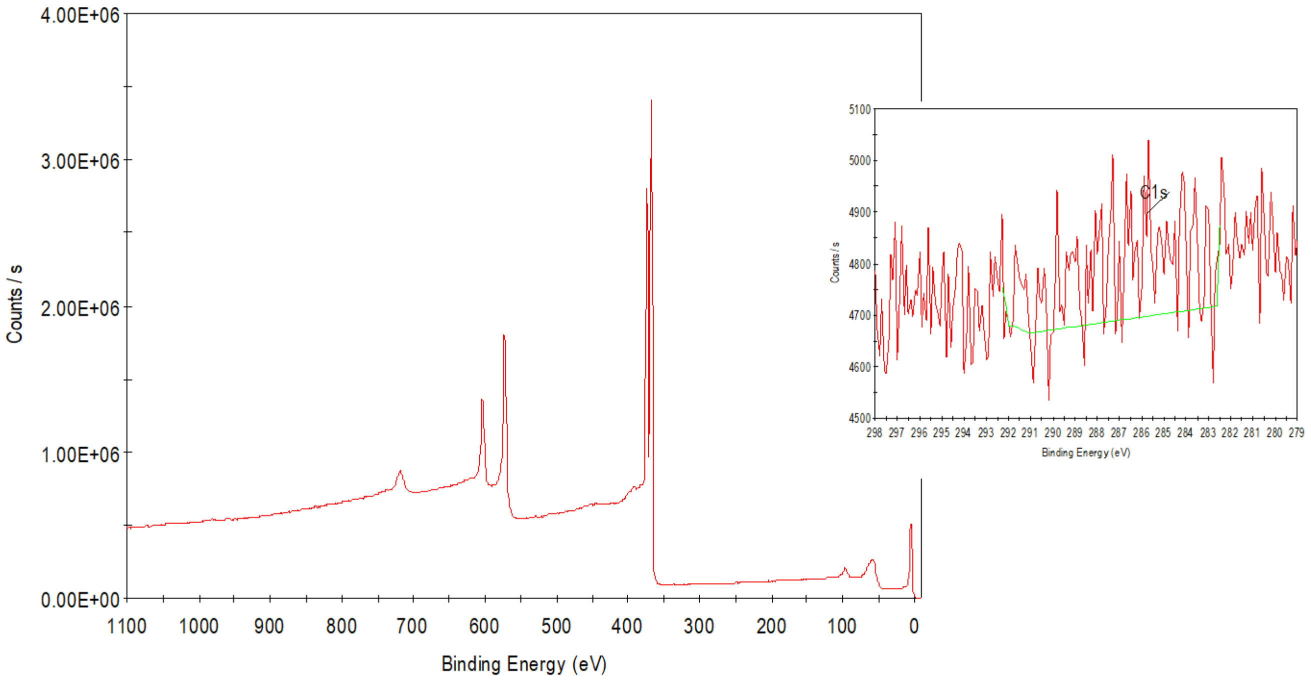

Figure S10. XPS survey spectra of the Ag/ITO/glass surface after the removal of adventitious carbon by argon sputtering. The Ag 3d region shows two peaks at 367.8 eV (Ag $3d_5/_2$) and 373.8 eV (Ag $3d_3/_2$), with a spin–orbit splitting of 6.0 eV, consistent with the presence of metallic silver at the surface.

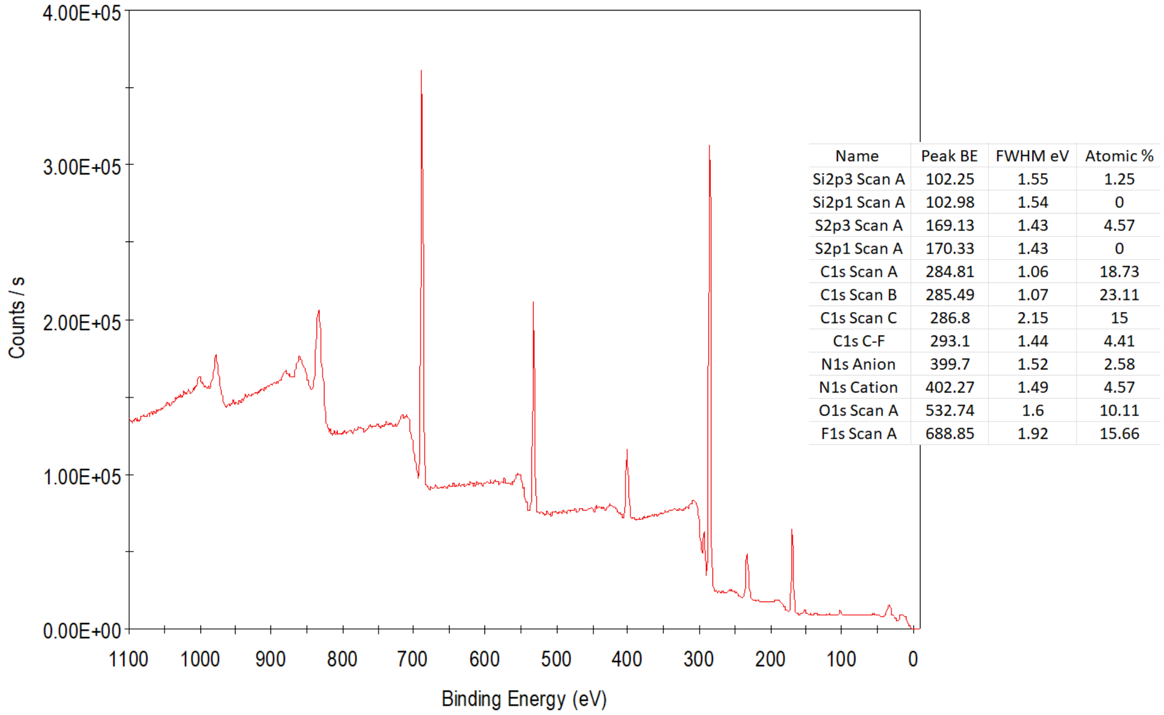

Name	Peak BE	FWHM eV	Atomic %
Si2p3 Scan A	102.25	1.55	1.25
Si2p1 Scan A	102.98	1.54	0
S2p3 Scan A	169.13	1.43	4.57
S2p1 Scan A	170.33	1.43	0
C1s Scan A	284.81	1.06	18.73
C1s Scan B	285.49	1.07	23.11
C1s Scan C	286.8	2.15	15
C1s C-F	293.1	1.44	4.41
N1s Anion	399.7	1.52	2.58
N1s Cation	402.27	1.49	4.57
O1s Scan A	532.74	1.6	10.11
F1s Scan A	688.85	1.92	15.66

Figure S11. XPS survey spectrum of a [$C_{10}C_{10}$im][NTf_2] film (400 ML) as-deposited by vacuum thermal evaporation (micro- and nanodroplets). The spectra show the characteristic signals of C 1s, N 1s, O 1s, S 2p, and F 1s. The detected species are consistent with the expected ionic liquid composition.

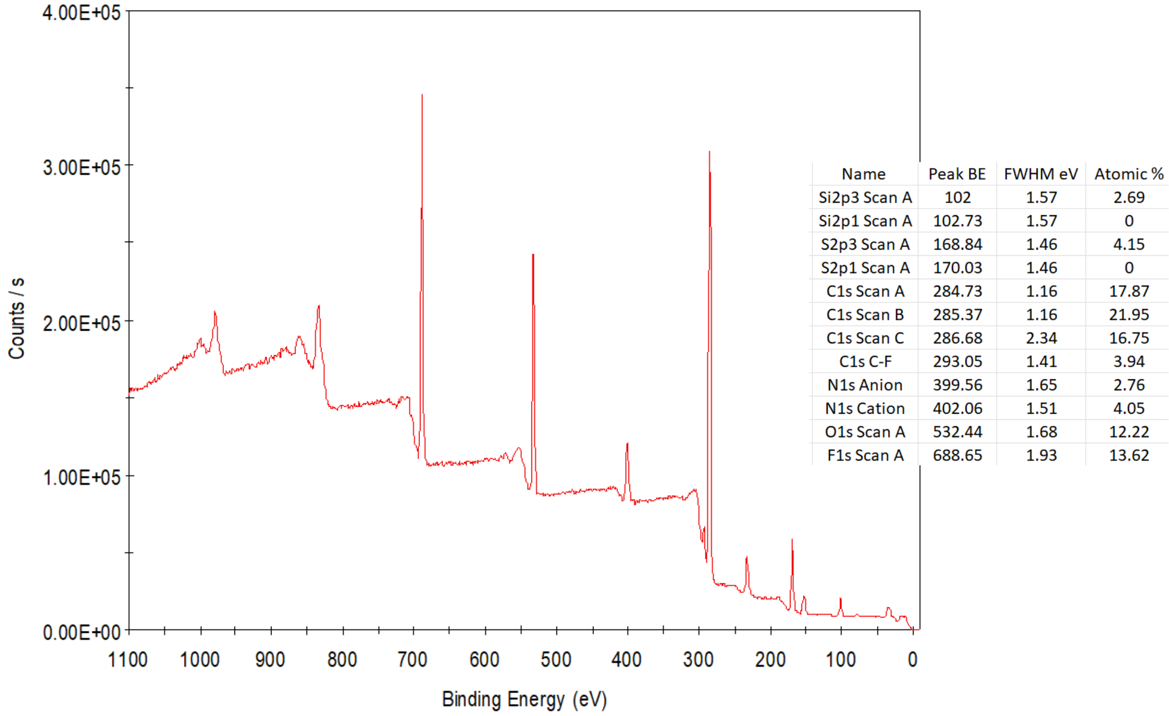

Name	Peak BE	FWHM eV	Atomic %
Si2p3 Scan A	102	1.57	2.69
Si2p1 Scan A	102.73	1.57	0
S2p3 Scan A	168.84	1.46	4.15
S2p1 Scan A	170.03	1.46	0
C1s Scan A	284.73	1.16	17.87
C1s Scan B	285.37	1.16	21.95
C1s Scan C	286.68	2.34	16.75
C1s C-F	293.05	1.41	3.94
N1s Anion	399.56	1.65	2.76
N1s Cation	402.06	1.51	4.05
O1s Scan A	532.44	1.68	12.22
F1s Scan A	688.65	1.93	13.62

Figure S12. XPS survey spectrum of a [$C_{10}C_{10}$im][NTf_2] film (400 ML) after argon plasma treatment (coalesced IL film). The spectra show the characteristic signals of C 1s, N 1s, O 1s, S 2p, and F 1s. The detected species are consistent with the expected ionic liquid composition.

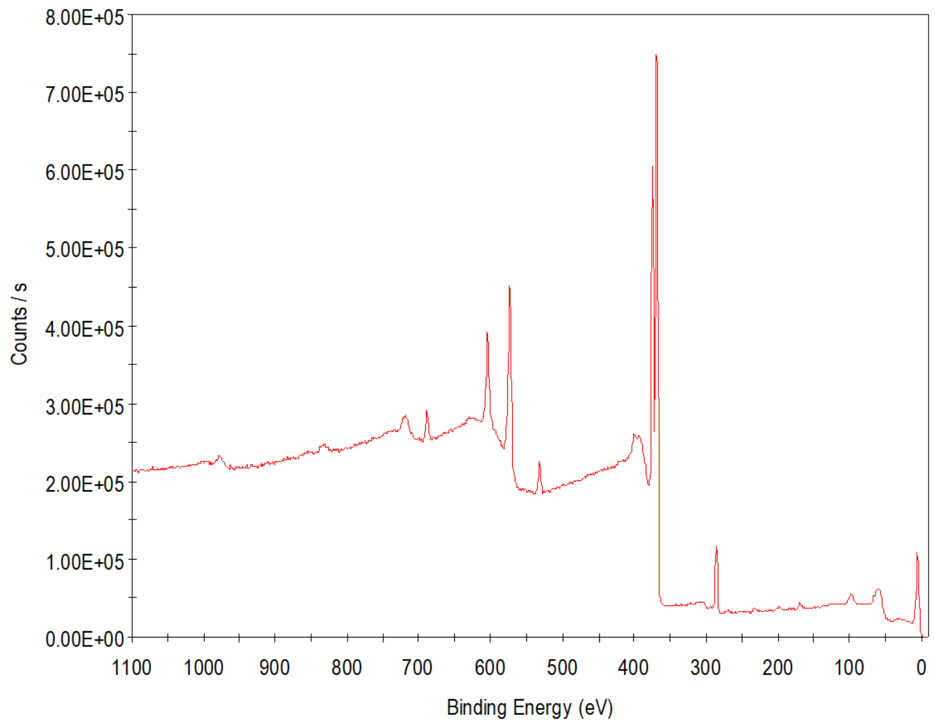

Figure S13. XPS survey spectrum of a [$C_{10}C_{10}$im][NTf_2] film (400 ML) after silver sputtering and 1 week of air exposure. The spectra show the characteristic signals of C 1s, N 1s, O 1s, S 2p, F 1s, and Ag 3d regions.

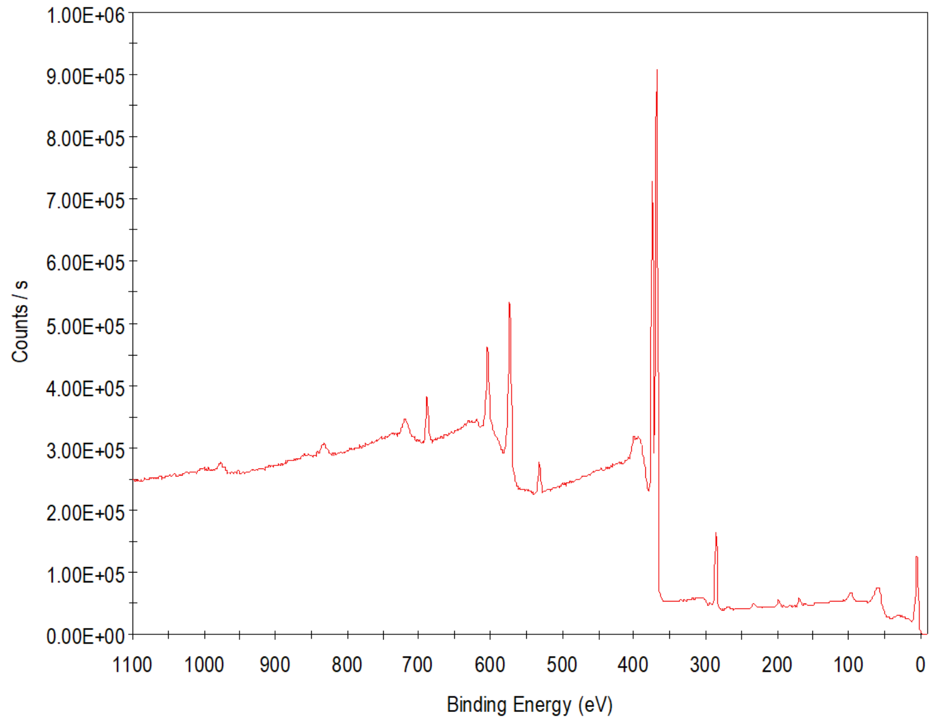

Figure S14. XPS survey spectrum of a [$C_{10}C_{10}$im][NTf_2] film (400 ML) after silver sputtering and 1 month of air exposure. The spectra show the characteristic signals of C 1s, N 1s, O 1s, S 2p, F 1s, and Ag 3d regions.

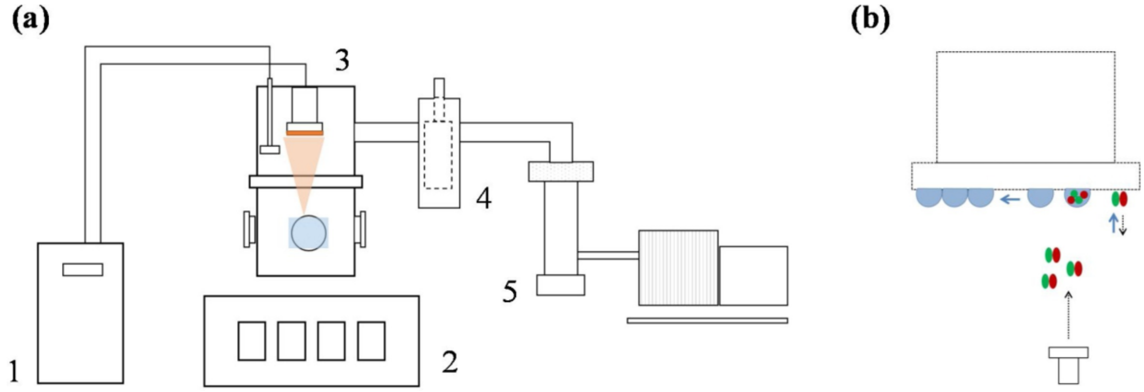

Figure S15. Schematic representation of the vapor deposition/vacuum thermal evaporation methodology: (a) ThinFilmVD apparatus (1 – cooling system, 2 – instrumentation box, 3 – vacuum chamber, 4 – N_2 (l) metallic trap, 5 – vacuum pumping system); (b) schematic detail of the PVD of ionic liquids by vacuum thermal evaporation from a Knudsen cell. More details: *Appl. Surf. Sci.*, 2018, 428, 242 and *J. Chem. Eng. Data*, 2015, 60, 3776.

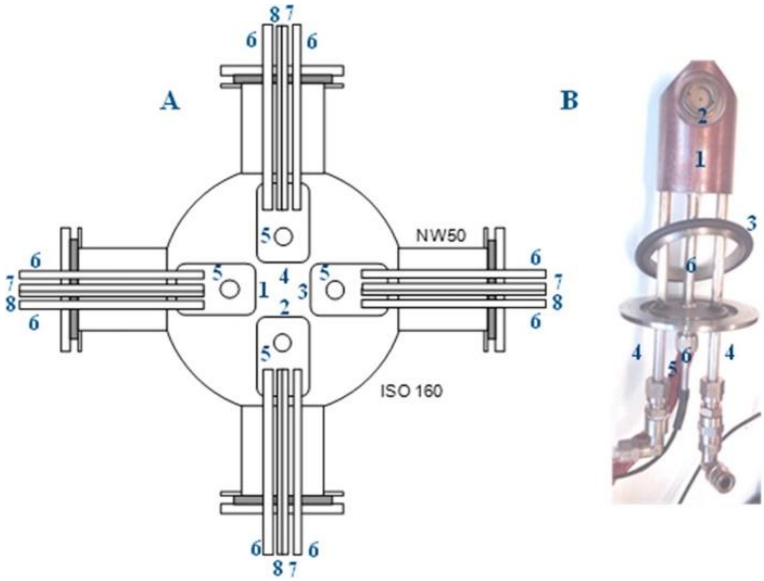

Figure S16. A – Schematic representation of the ovens of the Thin FilmVD apparatus: 1, 2, 3, 4 – individual copper ovens; 5 – cavity of the Knudsen cell screwing; 6 – air cooling tube; 7 – heater; 8, – Pt100 sensor; B – Image of an individual oven (top view): 1 – copper block; 2 – Knudsen cell; 3 – Viton O-ring; 4 – cooling system; 5 – heater; 6 – Pt100. More details: *J. Chem. Eng. Data*, 2015, 60, 3776.

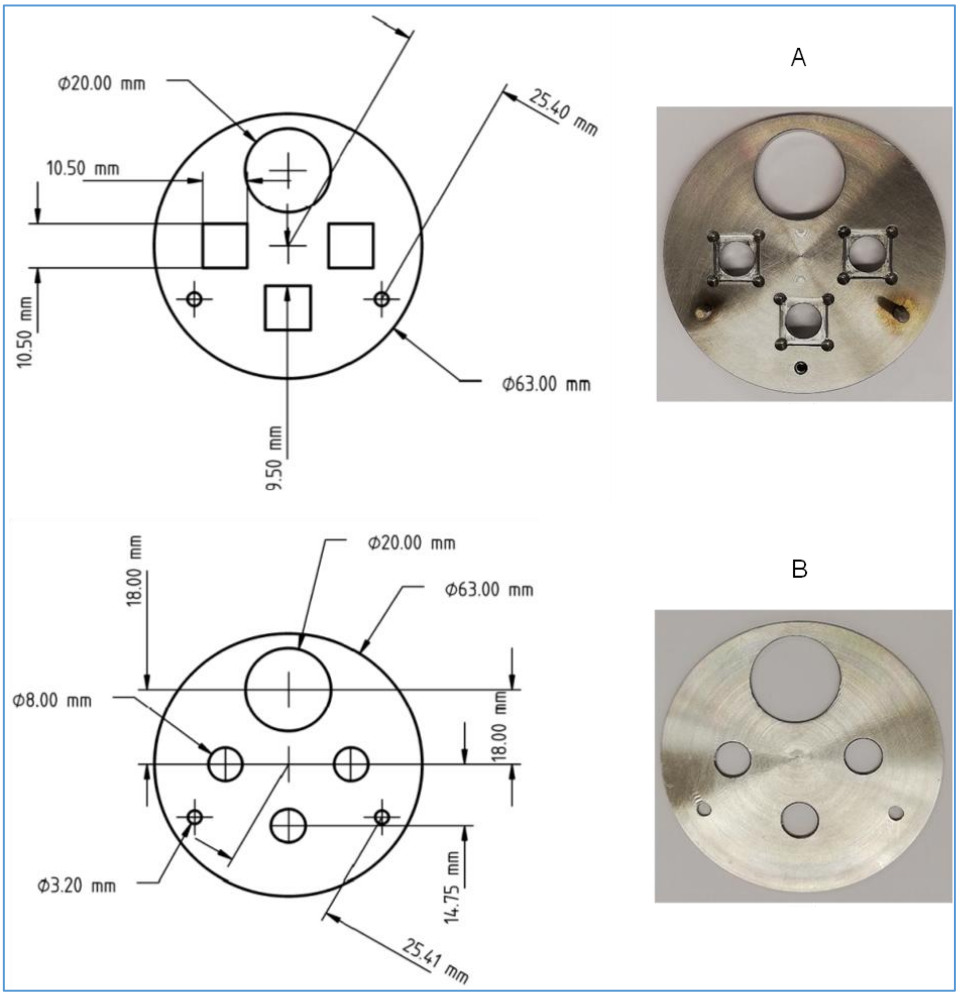

Figure S17. Schematic representation (left) and images (right) of the substrate support system. The support was used to deposit each ionic liquid (up to 3 film samples) on ITO/glass surfaces.

Table S1. Experimental conditions for the physical vapor deposition/thermal evaporation of each ionic liquid: effusion temperature ($T_{eff.}$); equilibrium vapor pressure (*EVP*); mass flow rate at the Knudsen effusion cell orifice (Φ (Knudsen cell)); mass flow rate at the substrate surface (Φ (QCM)) and corresponding deposition rate in Å·s^{-1}; geometric factor; deposition time; thin film thickness (nm and ML).

Precursor	$T_{eff.}$	*EVP*	Φ (Knudsen cell)	Φ (QCM)	Geometric factor	Deposition rate	Deposition time	Thickness
	K	Pa	μg·cm^{-2}·s^{-1}	ng·cm^{-2}·s^{-1}		Å·s^{-1}	min	ML
				**$[C_7C_7im][NTf_2]$ (400, 600 ML) / ITO-glass**				
**$[C_7C_7im][NTf_2]$** *M* = 545.69 g·mol^{-1} ρ = 1.225 g·cm^{-3} (a)	498.2	≈ 0.14 (b)	≈ 64	9.8 ± 1.3	≈ 2×10^{-4}	0.8 ± 0.1	≈ 75	400 (361 nm)
				11.0 ± 1.2	≈ 2×10^{-4}	0.9 ± 0.1	≈ 101	600 (543 nm)
				**$[C_8C_8im][NTf_2]$ (400, 600 ML) / ITO-glass**				
**$[C_8C_8im][NTf_2]$** *M* = 573.75 g·mol^{-1} ρ = 1.194 g·cm^{-3} (a)	498.2	≈ 0.10 (b)	≈ 47	9.5 ± 1.3	≈ 2×10^{-4}	0.8 ± 0.1	≈ 78	400 (371 nm)
				11.9 ± 1.2	≈ 3×10^{-4}	1.0 ± 0.1	≈ 93	600 (557 nm)
				**$[C_{10}C_{10}im][NTf_2]$ (400, 600 ML) / ITO-glass**				
**$[C_{10}C_{10}im][NTf_2]$** *M* = 629.82 g·mol^{-1} ρ = 1.152 g·cm^{-3} (a)	513.2	≈ 0.09 (b)	≈ 44	10.4 ± 1.3	≈ 2×10^{-4}	0.9 ± 0.1	≈ 72	400 (387 nm)
				12.7 ± 1.2	≈ 3×10^{-4}	1.1 ± 0.1	≈ 88	600 (581 nm)

(a) Density values at 298.15 K. (Ref. *J. Phys. Chem. B* **2013**, *117*, 10889–10897).

(b) Equilibrium vapor pressure at the effusion temperature. (Ref. *J. Chem. Phys.* **2014**, *141*, 134502).
